# Connected Text Reading and Differences in Text Reading Fluency in Adult Readers

**DOI:** 10.1371/journal.pone.0071914

**Published:** 2013-08-20

**Authors:** Sebastian Wallot, Geoff Hollis, Marieke van Rooij

**Affiliations:** 1 CAP Center for Cognition, Action and Perception, Department of Psychology, University of Cincinnati, Cincinnati, Ohio, United States of America; 2 Department of Psychology, University of Alberta at Edmonton, Edmonton, Alberta, Canada; 3 Interacting Minds Centre, Department of Culture and Society**,** Aarhus University, Aarhus, Midtjylland, Denmark; University of Leicester, United Kingdom

## Abstract

The process of connected text reading has received very little attention in contemporary cognitive psychology. This lack of attention is in parts due to a research tradition that emphasizes the role of basic lexical constituents, which can be studied in isolated words or sentences. However, this lack of attention is in parts also due to the lack of statistical analysis techniques, which accommodate interdependent time series. In this study, we investigate text reading performance with traditional and nonlinear analysis techniques and show how outcomes from multiple analyses can used to create a more detailed picture of the process of text reading. Specifically, we investigate reading performance of groups of literate adult readers that differ in reading fluency during a self-paced text reading task. Our results indicate that classical metrics of reading (such as word frequency) do not capture text reading very well, and that classical measures of reading fluency (such as average reading time) distinguish relatively poorly between participant groups. Nonlinear analyses of distribution tails and reading time fluctuations provide more fine-grained information about the reading process and reading fluency.

## Reading and Text Reading

Reading, together with writing, is one of the hallmark activities that distinguish humans from other animals. Language processing has been the topic of extensive study in the cognitive sciences. Of particular interest to this paper are research efforts focused on the facet of reading. Reading research has taken varied approaches, from scrambled word reading [1], to non-word-symbol insertion into texts [2] or the investigation of cross-modal effect of spoken on written language perception [3]. However, one fact catches the eye: Reading is almost always studied either in terms of single words (standard word naming or lexical decision tasks), word pairs (priming tasks), or single sentences, a hand full of sentences at most.

Rayner and Pollatsek [4] survey the approaches that cognitive scientists have taken to investigate the process of text reading, and most of the experimental setups use no more than two sentences, perhaps holding at about twenty words altogether. The same is true for an overview article by Clifton and Duffy [5], where studies of ‘text’ reading encompassed eight sentences at the most. The layman might be puzzled by this sparsity, but this very sparsity has been adopted by psycholinguists and other reading researchers for good reason: consecutive presentations of as few as two words in a row already result in complicated carry-over effects.

For example, a key-press response in a simple reading task, to indicate that the word *pepper* is indeed a word (with respect to English spelling) will be about 48 ms faster (on average) if *pepper* is preceded by the word *salt* (compared to a control condition that precedes pepper by an unrelated word such as *loan* – [6]). This is a large effect, given that a single word is easily read within about 200 ms from first sight [4] However, if *salt* is presented twice in succession, in the same task, just before *pepper* appears, the large facilitation effect vanishes [6], [7]. If this was merely an isolated oddball finding then it might be of little consequence, but all simple reading tasks reveal such complicated patterns of interaction among the factors that reading scientists study (see [8], [9] for reviews and discussions).

Slight variations in laboratory tasks can result in large changes to reading performance [10]. Nonetheless, the default assumption in almost all reading research has been that the impact of a contributing factor to reading performance, whether the property of a text or of a reader, will be proportional to its magnitude. A less skilled reader should require proportionally more time and effort to read the same text, and a more difficult text should require proportionally more time and effort to read. But reading may comprise a heterarchy of overlapping and interacting capacities, such that different combinations may even compensate for deficiencies, insofar as reading speed or comprehension are concerned. This is well illustrated by an example given by Rayner and Pollatsek [4]. It is generally found that very fast readers ‘skim’ though text, exhibiting fewer fixations during reading, which in turn can have an impact on text comprehension. However, when for example a political leader reads through the first few pages of a daily newspaper, he or she might do so at the limit of speeded reading while maintaining a high level of content knowledge, simply because he or she was directly involved in most of the events portrayed on the first pages of the newspaper.

Accordingly, psycholinguists have come to understand that there exist complicated relationships between reader and text properties, but also even the most basic word descriptors, the lexical variables (such as word frequency), that putatively capture the cognitively salient features of words. Hence, caution is warranted when one confronts the scientific investigation of more extended text units, since one will face the above-mentioned complications all at once [11], [12].

Another reason why the investigation of individual words and sentences is so prominent is that they seem to encapsulate the essentials of written language: Words contain the basic meanings and lexical features of written language, while sentences supply the syntactical features, which can be sufficiently tabulated within a single sentence unit. Following this logic, the investigation of single words and single sentences contains the potential to uncover the basic features and rules of written language perception, which should be the principal basis for all reading performance [13].

In an idealized lexicon, these constituents play a central role and are elaborately described to include meanings, spellings, pronunciations, and even the possible uses in sentence constructions [14]. The constituents are elementary units and their use in conjunction with grammatical rules depends on their unchanging character. They do not possess any interesting dynamics of themselves and their entries in the lexicon should not depend on any contextualization apart from what is already specified in their representation.

The pragmatic reason of manageability and the theoretic assumption that words or sentences are the basic constituents of reading behavior lead to the presumption that a characteristic scale of reading performance exists, which might be found on the word and/or sentences level. Because of this point, and the difficulties outlined above, few studies have concerned themselves with either reading on the text level or carry-over effects between sentences in a text [15], [16].

However, new statistical methods have been introduced into the analysis of cognitive performance that allow for, and motivate a different approach to text reading research: Instead of focusing on the quantification of local contributions to observed reading performance, such as the effect of a word’s frequency on its reading time, these analysis techniques seek to quantify bigger parts or even whole episodes of cognitive performance (for a summary and tutorial see [17]). Wallot and Van Orden used these methods to analyze the variability, stability and interconnectedness of reading performance in a self-paced reading task, where participants reveal every new piece of text with a button press [18], [19], [20]. Could show that the reading dynamics of longer text chunks (i.e., sentences) were much more informative compared to shorter text chunks (i.e., individual words) that are commonly used in self-paced reading tasks [18], [20]. Furthermore, they found that nonlinear statistics were more sensitive in distinguishing between more and less fluent readers [20]. These first results motivate a more thorough comparison of more traditional and nonlinear metric of the text reading process.

Following these preliminary studies, the goal of the current article is to demonstrate how online reading performance of long connected text (i.e., over 10,000 words) can be analyzed using multiple statistics and reading metrics. We will show how the outcomes of the multiple utilized analysis techniques can be used to inform each other, and give a more detailed picture of text reading performance. In order to do so, each analysis is performed, the results are presented, implications are discussed, and successive pieces of information are incorporated into the interpretation of recorded the text reading data. Two novel data analysis techniques, Fractal Analysis and Recurrence Quantification Analysis are introduced in more detail to the interested reader. Both methods quantify the structure of the *evolution* of the reading process, compared to summary measures that collapse across time (e.g., central tendencies or dispersion measures). We want to bring this analysis strategy to immediate use to investigate the effect of different kinds of reading fluency (habitual and situated) on text reading performance, as described in the next section.

### Reading Fluency

To be called a fluent reader, a person must be able to understand written text and readily comprehend connected text. This is one prerequisite of reading fluency. Reading fluency is not just skilled reading in the sense of understanding: Fluency also implies an effortlessness in the act of reading, so that written text is understood by the reader easily; fluent readers can progress through a text quickly and flexibly. A minimal definition of reading fluency might encompass the terms comprehension, effortlessness, flexibility, and–superficially–reading speed [18].

Since fluency develops over the course of each individual’s lifetime, initially co-evolving with basic reading skill, investigations are often necessarily confined to a semi-experimental approach where participants’ reading fluency is estimated from prior information (reading test scores, or age). However, one experimental manipulation of reading fluency has been proposed by Samuels [21], which is the method of repeated readings.

Here, a participant is asked to read the same text more than once, and the underlying rationale is that the second reading will have increased the participant’s reading fluency for this text by providing her or him with an increase of general knowledge about the text, with increased familiarity in its specifics, and strong expectations about its content.

Regarding reading fluency, our specific questions are how differences in reading fluency map onto reader text reading performance, and how the two kinds of manipulations of reading fluency–differences due to the first and repeated reading, as well as differences due to different abilities between two participants groups–contrast with each other.

### The Self-paced Reading Task

In a self-paced reading task, participants read longer text units, consisting of one or several sentences worth of words, advancing themselves through the text by pressing a response key to reveal every new text chunk. The text chunks are usually single words that make up a piece of text. The intervals between two consecutive key-presses are then interpreted as an estimator of the reading time of a text unit [4].

We picked a science fiction short story (which is described in more detail in the method section) to minimize advantages due to topic knowledge. The collected data are then analyzed in several sections using different psycholinguistic measures and analyses, aiming at giving a detailed picture of text reading performance, illustrating the utility of the different analyses and showing how their individual results inform each other. In a way, the different statistics we employ can be seen as different ‘observers’, each reporting a different detail about the reading performance in question.

According to Samuels [21], repeat readings improve fluency by increasing content knowledge, improving familiarity with document specifics, and providing expectations about future content. Such fluency can be gained even within the span of single reading; most writing – whether fictional or nonfictional – foreshadows or explicitly states what the reader can expect to find in the rest of the text. We can expect that within the span of a single reading, individual reading performance will change as readers develop stronger expectations and accrue more situational knowledge about the text they are reading.

Compared to novice readers, we might also expect expert readers to show fewer effects for lexical variables, progress within a text, and repeat readings of the same text. Presumably expert readers have more knowledge and experience about the process of reading, allowing them to more quickly develop fluency within a specific document. However, the role of lexical variables play for reading skill has never been investigated in text reading.

Finally, the extent to which lexical variables such as word frequency affect reading performance in full text reading is informative about how much fluency and content knowledge is relevant to the process of reading. Lexical effects for single-word reading are prone to complicated contextual effects [8], [9], which are driven by providing readers with expectations or priming of what might follow. Since most writing provides heavy expectations to readers, we hypothesize that these higher-order contextual effects (and the reader’s ability to pick up on to them) will wash out individual lexical effects if the contextual effects are strong enough.

## Method

### Ethics

The study was approved by the institutional review board of the University of Cincinnati (IRB protocol number 07040402E). Before the beginning of the study, participants were presented with a written consent form and written consent was obtained from each individual.

### Participants

Forty-nine students of the University of Cincinnati participated in the reading study. Half of them (*n* = 25) were undergraduate students in their first or second year, the other half were graduate students of English Literature and Psychology. Of the undergraduate students, 14 were female (58.33%) with a mean age of 21 years (ranging from 18 to 39 years). Of the graduate students, 13 were female (54.16%) with a mean age of 28 years (ranging from 21 to 43 years). All were native speakers of English and all had normal or corrected-to-normal vision. Participants were compensated for their participation in the form of class credit and money (participants were paid 20 USD for their participation).

### Materials and Apparatus

The text used in this study was a science fiction short story entitled ‘The Arles Complex’ by Louis P. DeGrado [22]. This story describes fictional intergalactic politics of the race of the Arelians, who were nearly wiped out in a previous conflict with another civilization. The story consists of 13930 words, 1696 phrases, and 1042 sentences. Each phrase was a sequence of words demarcated by any punctuation (comma, period, colon, semicolon, parenthesis, question mark, exclamation mark, or dash). The average word length in the story ‘Arelis Complex’ is 4.56 letters (*SD* = 2.38), and the average sentence length is 13.36 words (*SD* = 7.89), lying well in the range of standard English prose [23], [24]. The Flesch-Kincaid index of readability assigned the text a score of 6.0, indicating that its difficulty is appropriate for early 6th grade readers. Readability formulas are not without problems [25], but we reasoned that the text as such should make an easy reading for college students at any stage.

The text was displayed on a standard 16-inch diameter computer monitor in the Times New Roman font (13.5 pt.). Responses were collected using the space bar of a standard computer keyboard. The text presentation was controlled by a custom MATLAB Psychtoolbox [26] script that displayed text units and recorded key-presses. After reading the story, each reader was tested to assess story comprehension and memory. The test required a written summary of the story plot, indicating characters and their roles in the story, as well as a multiple-choice sentence completion task. Items for the sentence completion task covered the entire story, and only data of participants who answered at least 9 out of 10 multiple-choice questions correctly were included in the analysis. All but one participant did so successfully.

### Procedure

**Figure 1:**
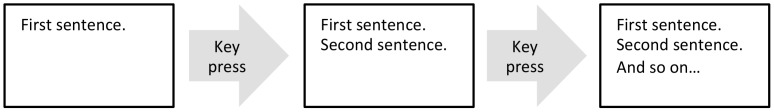
Illustration of the reading task with sentence units. Participants revealed each new text unit by pressing a space bar. Text would build up on the screen, line by line, until the whole screen was filled with text. When the whole screen was filled with text and the participant hit space once more, the screen would blank and the next text unit (word, phrase, or sentence) would appear in the upper-left corner.

After obtaining written consent, participants were asked to read a short story, displayed on a computer monitor (either word-by-word, phrase-by-phrase, or sentence-by-sentence, depending on the condition). Participants were instructed to reveal each new text unit (word, phrase, or sentence) by pressing the space bar, so that text would build up on the computer monitor as illustrated in Figure 1. Participants were asked always completely read a text unit before revealing a new one.

Before starting the reading task, participants were informed that they would have to complete a comprehension test after reading the story. Participants were informed that merely reading of the story would be sufficient to answer all questions successfully; deliberate memorization of specific parts of the story would be unnecessary. This advice was given to ensure participants were motivated to read, and did not to simply press the response key [16]. Half of the participants returned for another reading session and were instructed to re-read the same text again.

## Results

### Overview

We applied a variety of conventional and novel statistical techniques to analyze the results of the self-paced reading task described above. First, we report average reading times, followed by an analysis of distribution properties or reading times. Then, we turn to an analysis of classical lexical variables (word frequency, word length, word co-occurrences), as well as a novel lexical variable, text redundancy, which is quantified using Recurrence Quantification Analysis. Finally, we conduct nonlinear time-series analysis of reading times (Recurrence and Fractal analysis).

For each analysis, we report the associated statistics and then briefly discuss the results. Follow-up analyses are conducted where needed. The general discussion at the end of the paper will bring together all the information obtained from the analyses and summarize what has been learned about text reading, the reading task, and differences in reading fluency.

For the analysis of the reading data, each measure was first subjected to a between-subject analysis of variance with the factors text unit (word vs. phrase vs. sentence), reader group (undergraduate students vs. graduate students), and number of reading (reading once vs. repeated reading). However, almost all of the analyses yielded statistical interaction effects including the factor text unit (i.e., the direction of the effects observed was heavily dependent on whether the text was presented word-by-word, phrase-by-phrase, or sentence-by-sentence). In order to properly investigate the effects of reader group and number of readings, all analyses were broken down by the factor *text unit* when interactions between the factors were observed.

### Average Reading Times

**Figure 2:**
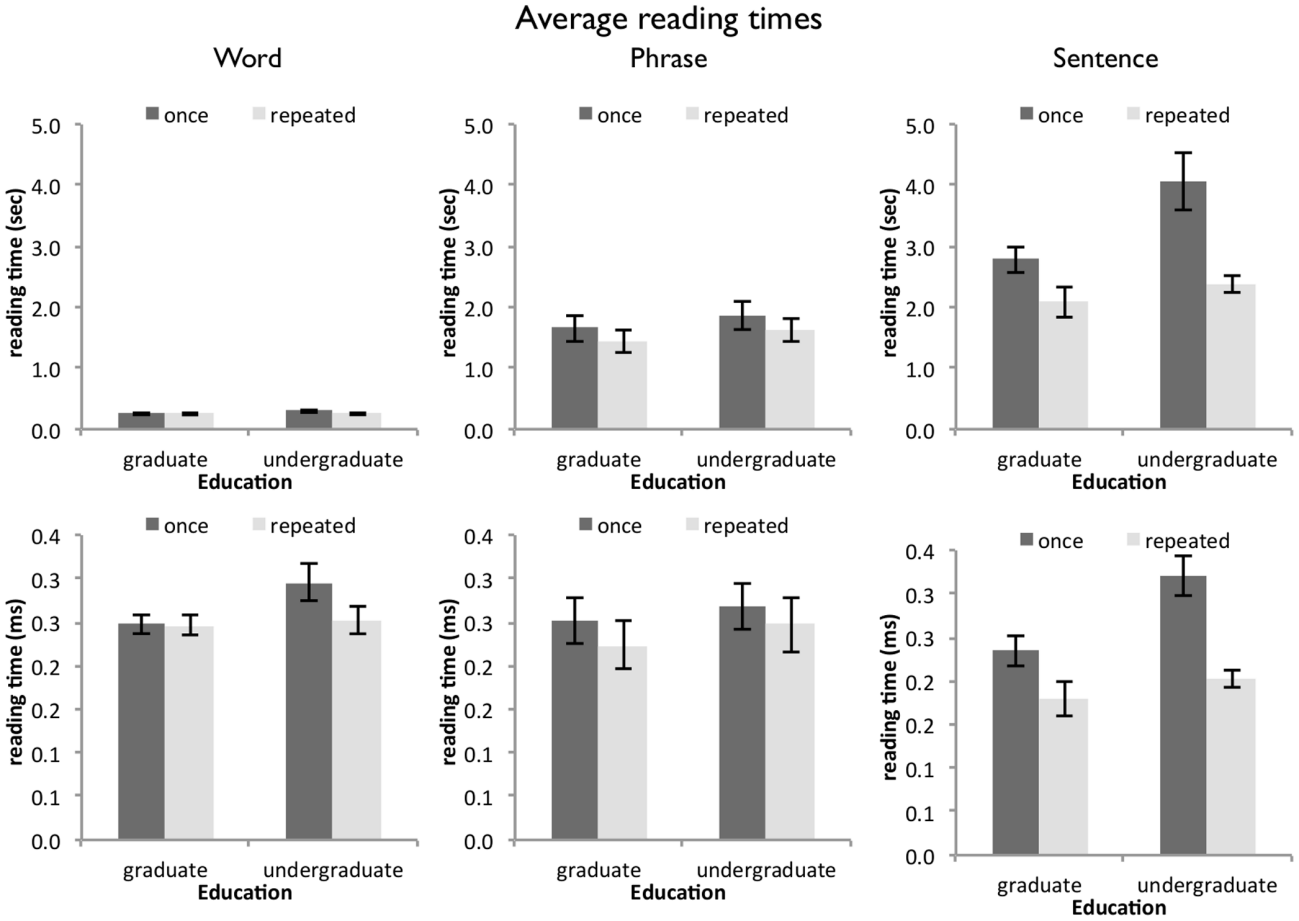
Average reading times for the three text units (words, phrases, and sentences) by reader group and repeated reading. The upper panel shows reading times for word (left column), phrases (middle column), and sentences (right column). As can be seen, it takes longer to read bigger text units. Also, as can be seen in the upper-right panel, sentence reading times are faster for graduate readers compared to undergraduate readers, and generally decrease with repeated reading as well. The lower panel shows the same data, when the reading times for phrases and sentences were scaled down to the number of words. Average word reading times do not differ reliably between the three text-unit conditions.

As reading fluency is generally assumed to result in a higher reading speed [27], we expected average reading times for graduate students to be faster compared to undergraduates, as we assumed that graduate students are more experienced readers. Also, we expected readers who read the story repeatedly to read faster due to the increase of familiarity with the text [21]. Figure 2 summarizes the results.

Average reading times in the sentence unit condition were longer for undergraduate readers (*F*(1, 28) = 6.21, *p*<.05) compared to graduate readers. Also, average reading times decreased with repeated readings (*F*(1, 28) = 15.60, *p*<.001). No other effects were apparent (all *F*<3.00).

### Information Obtained from Average Reading Times

For word units, it seems that all participants performed at ceiling (on average at around 240 ms), close to the speed of simple reaction times [28]. Proficient readers under ‘normal’ circumstances (i.e., who have a whole page full of text available to them) however, usually read even faster, at 200 ms per word [4]. It seems plausible that the responses collected from word-unit presentation are not valid estimates of individual word reading times. Wallot and Van Orden [18] suggested that performance of word-by-word self-paced reading is delimited by the ability of the reader to press the response key as fast as possible. That is, the response execution via the hand is inherently slower than the possible speed of eye-movements during reading.

This also makes for an interesting contrast to the response times observed in other tasks that are commonly used to investigate reading performance, such as lexical decision or word naming (where response times are in the range of 600 to 900 ms [29]), which occur on a different time scale than text reading. We will discuss in how far sequential priming effects and other text characteristics could be responsible for these observed differences later on in the analyses of word co-occurrences and redundancies.

Like word reading times, phrase reading times do not reveal differences between reader group or repeated reading. Also, the average time to read a word in the phrase condition was not appreciably faster than the average time to read a word in the word-unit condition. This is surprising, given that the average phrase contained 8.21 words and might – in principle – have allowed for faster reading times, was the need for key-presses between words was greatly reduced. At this point, it remains unclear why phrases are not read faster with repeated reading, but we will return to this result as we present further analyses of the reading data.

Finally, the sentence unit condition revealed the expected differences between reader group and repeated reading. Repeated reading facilitates reading performance, perhaps by virtue of increasing reading fluency of the reader [21], at least for a particular text. As observed previously [18], [19], sentence unit reading seems to be the more sensitive mode of text presentation, at least as it pertains to the two reader groups selected here.

**Figure 3:**
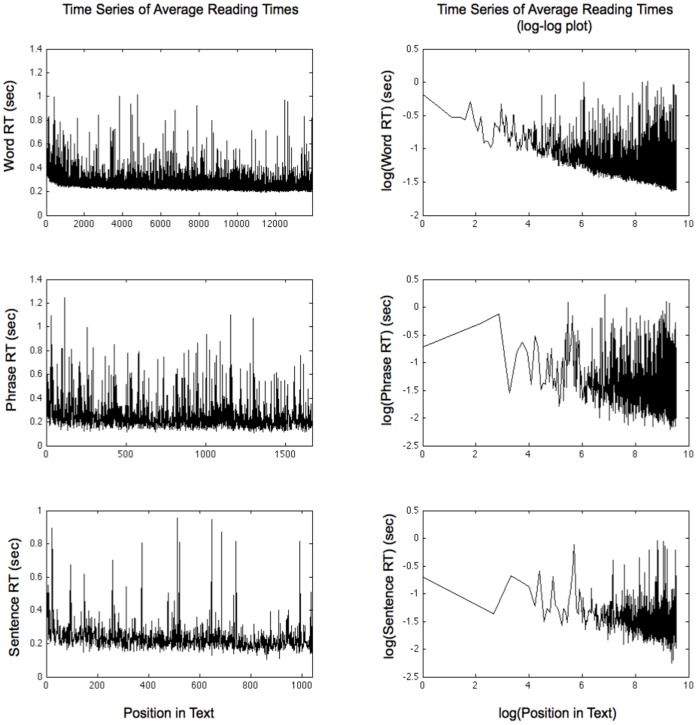
Time-series of average reading time for words (top row), phrases (middle row), and sentences (bottom row). The left column presents the time series of mean reading times (standardized by the number of words for the phrase and sentence conditions). Word reading times become faster as story reading progresses, especially during the first 500–600 words (which equals the first 60–70 phrases or the first 40–50 sentences). The right column shows log-log plots of the time series of mean reading times: mean log reading times decrease linearly with log position of the word, phrase, or sentence in the story. That is, word reading times decrease as a power-law function of their position in the story. The slope of a linear regression line fitted to the log-log reading times estimates the magnitude of the decrease, which is −0.09 for words, −0.10 for phrases, and −0.09 for sentences.

Compared to our data, most other studies that employ self-paced reading reported average reading times per word that range from 400 to 600 ms [4], [30] - reading times at about two to three times as high as the ones we observed. These diverging findings may be reconciled, when we look at the change of average word reading times for word-units, phrase-units, and sentence-units over the course of reading (Figure 3).

There is a marked decrease in mean reading times over the first 500 to 600 words (which equals the first 60 to 70 phrases or the first 40 to 50 sentences). When we look at the length of the texts used in [30], for example, where participants read passages of 130 words, the average word reading times was 462 ms (*SD* = 233 ms). When we calculate the average word reading times for the first 130 words in our study, we can see that they fall within the region of the start-up transient, at which we observe average reading times of similar magnitude (*M* = 487 ms, *SD* = 354 ms for word-unit presentation, *M* = 499 ms, *SD* = 344 ms for phrase-unit presentation, and *M* = 448 ms, *SD* = 266 ms for sentence-unit presentation (when read for the first time)).

A closer examination reveals that the downward trend in reading times continues throughout the entire reading session. This decrease in reading time can be captured by a power-law function of the position at which a word appears in the text. Figure 3 shows plots of the time series of average reading times on a double logarithm scale (log-log), where the logarithm of the word reading time is plotted against the logarithm of the position of that word in the text. When a least-square regression line is fitted to the log-log plot of reading times and word position, the slope of that line estimates the strength of the decrease in reading time, which is −0.09 for words, −0.10 for phrases, and −0.09 for sentences.

Reading times that change during text reading as a power-law function of position indicate that reading performance differs systematically as a function of the location of a particular word in the text. Such power-law functions of trial or item position have also been observed in learning tasks [31] and are indicative of a scale-free process, where task constraints – or, as in our case, text-content constraints – accumulate and shape task performance. That is, reading of a particular word or sentence is not just a function of its local properties, but also a function of all of the text that precedes it.

The log-log plots of word position versus mean reading time capture a unifying property of the text reading process throughout the three text-unit sizes. However, at this point it remains an open question what characterizes the differences in performance between the reader groups and repeated readings, or why these differences are mostly observed in the sentence unit condition. The observation that average reading times decrease as a power-law function suggest the importance of accumulated constraints over time. Information on how the constraints interact with performance is often inherent in the shape of distributions of response times [30]. Reading times usually do not conform to a Gaussian distribution but their shape varies along a continuum between lognormal and inverse power-law distributions. Hence, analysis of the tails of distributions can reveal additional information about how changes in means come about, or how constraints impact the measured reading performance [31], [33].

### Analysis of Distribution Tails

The shape of the reading time distributions can reveal information about the underlying dynamics of the reading process**.** Previous studies investigating cognitive performance suggest that tasks with few constraints produce response time distributions, which conform to a power-law shape. Tasks with a high degree of constraints on performance, on the other hand, produce response time distributions, which conform more to a shallow power-law or lognormal shape, decreasing the heaviness of the distributional tails [33]. Experience in a task is equivalent to accumulation of constraints and we expect that repeated reading of a text leads to less heavy tails of the reading time distributions. According to the same logic, differences in reading experience – assuming that experience and repeated reading are both a manipulation of reading fluency – should have similar effect on reading times, leading to less heavy tails in the reading time distribution.

**Figure 4:**
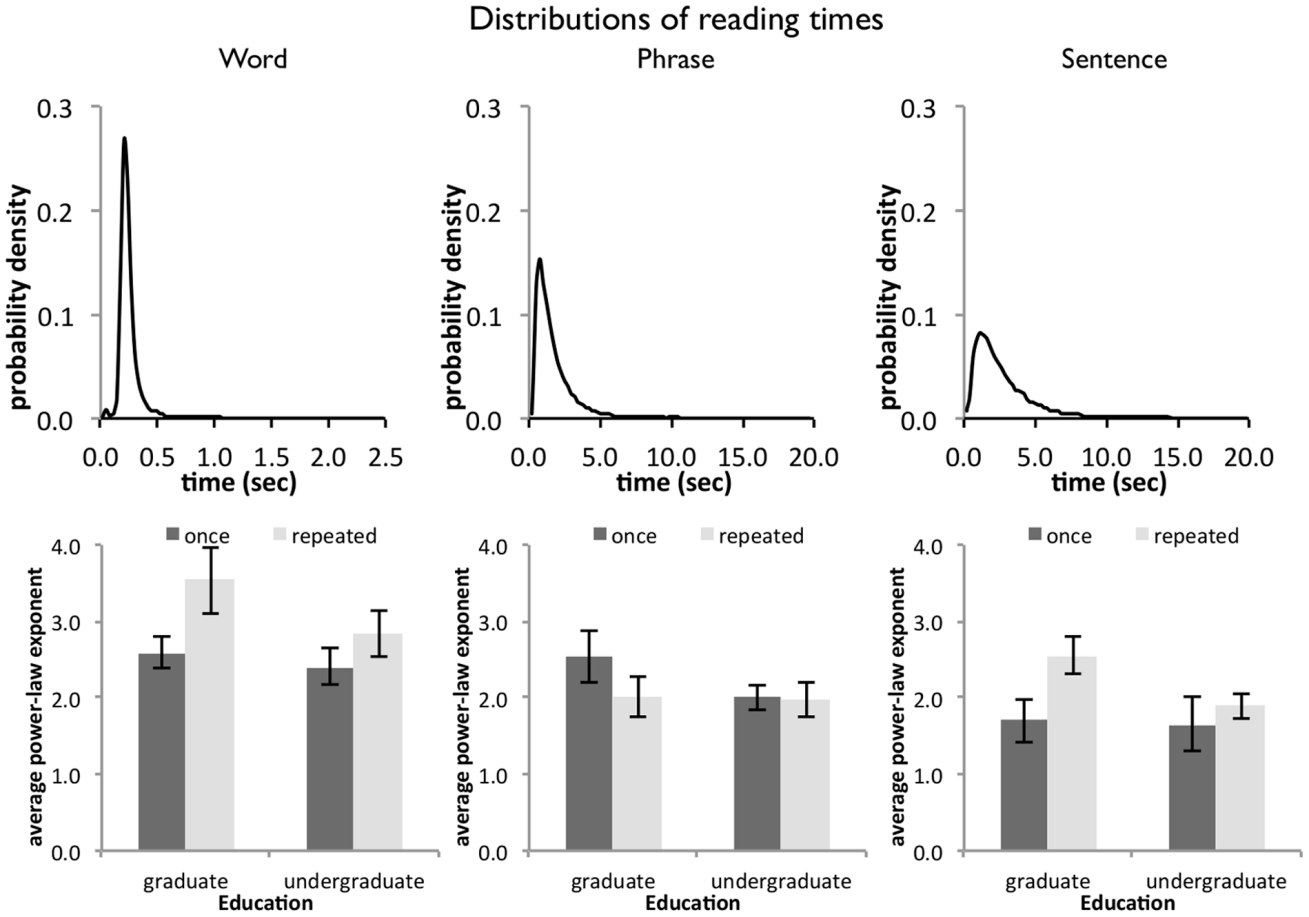
Distribution properties of reading times. The upper panel displays the aggregated response time distributions for the three text units, words, phrases, and sentences (in that order). Note that the x-axes for phrases and sentences reach from 0 to 20 seconds, while the x-axis for word reading times reaches from 0 to 2.5 seconds. The lower panel displays the average α exponent of the response time distributions by text unit, reader group, and repeated reading. The distribution tails for word and sentence reading times are indeed steeper for repeated reading than for reading once.

To obtain a statistic on the ‘heaviness’ of the distribution tails, the tail of the distribution is plotted on a log-log scale, and the slope of a fitted least-square line gives an estimate of the exponent [32], where the slope −*S* = power-law exponent. Figure 4 shows the aggregated distributions of reading times for word-by-word, phrase-by-phrase, and sentence-by-sentence presentation, as well as the power-law exponents of the slopes of the response time distributions associated with each reading condition.

In the word unit condition, the tails of the distributions tended to become steeper with repeated readings (*F*(1, 28) = 4.13, *p* = .052). Similarly, in the sentence unit condition, the tails of the distributions also tended to become steeper with repeated readings (*F*(1, 28) = 4.12, *p* = .052). No other effects were apparent (all *F*<1.28).

### Information Obtained from the Analysis of Distribution Tails

The analysis of distribution tails highlights the effects of repeated reading on the distribution of reading times: When the text is read repeatedly, the power-law exponents of the tails increase, which means less heavy tails. Hence, there are fewer very long reading times with repeated reading, at least in the word and sentence unit conditions, compared to the first reading. Again, the distributions of reading times of phrase-unit reading are unaffected by either repeated reading or reader group.

Changes to a distribution’s tail impacts the calculation of other moments, such as the mean. Instead of using the mean to estimate the central tendency of reading times, it may therefore be more warranted to consider the mode of the distribution as a proper estimator of central tendency [34].

**Figure 5:**
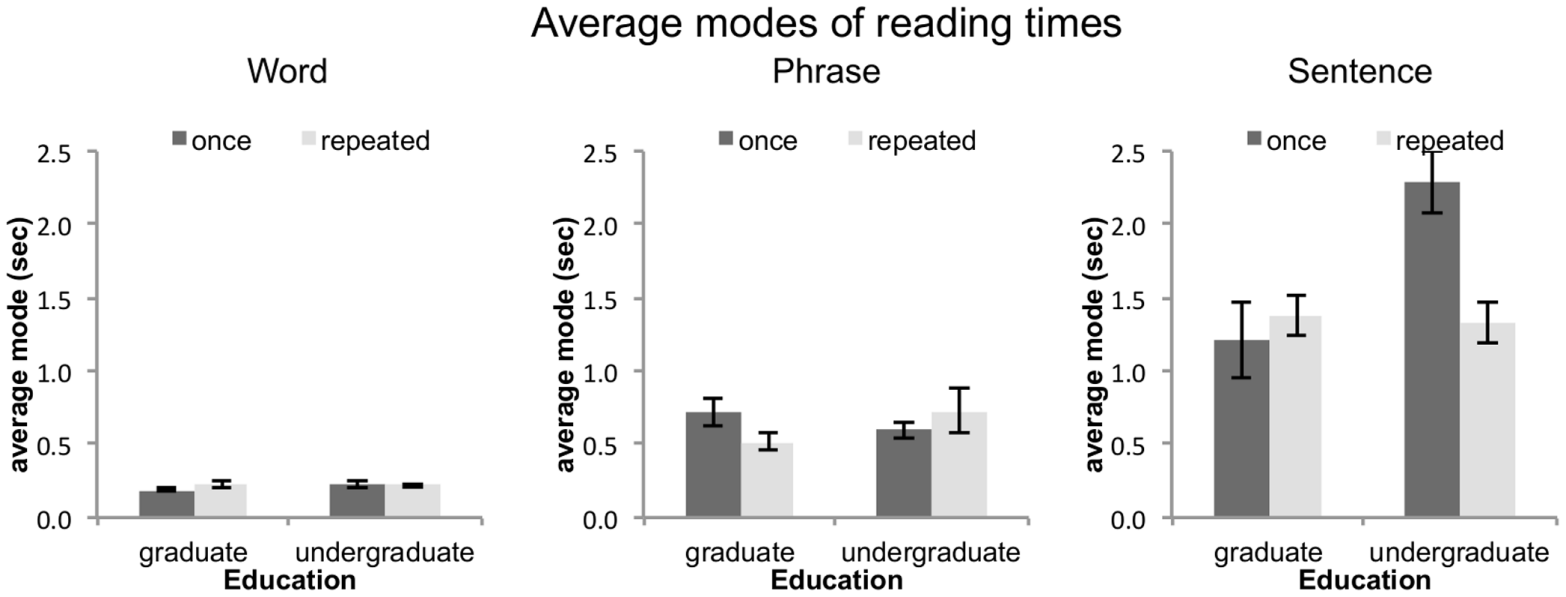
Modes of the reading time distributions for words, phrases, and sentences. The modes of sentence reading times decrease for undergraduate readers, while no such change is observed for graduate readers. There were no changes in the modes of word- or phrase-unit reading times.

Figure 5 displays the modes of the reading time distributions by text unit, reader group, and number of readings. There are no changes for the modes of the reading time distributions for word and phrase reading times (all *F*<3.01). However, there is a main effect of reader group on the distribution modes of the sentence reading times (*F*(1, 28) = 5.25, *p*<.05), which is qualified by an interaction between the factors repeated reading and reader group (*F*(1, 28) = 6.35, *p*<.05): Only undergraduate readers benefit from repeated readings, in the sense that the central tendency of their reading time distributions shows a decrease with repeated reading.

Re-analysis of the mean reading times, controlling for the slopes of the distributions confirmed this result: We now observe an interaction between repeated reading and reader group (*F*(1, 28) = 4.57, *p*<.05), indicating that the gain in average reading times with repeated reading is due to a decrease in the undergraduate readers’ reading times (there are no changes in the means for word or phrase unit reading, all *F*<2.66).

A reduction in extreme reading times suggests that repeated reading constrains reading performance – for example, reading times for difficult words or surprising passages of the text have less of an impact on the key press performance. Reading times are generally closer to the central tendency of the distribution.

The changes in the sentence unit condition are more complex. There is a clear change in the central tendency, indicating that undergraduate readers gain overall speed with repeated text reading, while no such change is observed for graduate readers. Furthermore, there are effects of repeated reading for both reader groups on the steepness of the distributional tails: response time variability for both groups is reduced as extreme response times moved closer to the distribution’s central tendency.

To illuminate the nature of the changes observed in reading performance, we investigate the relation of lexical variables to reading times in the next section. In particular, we look at the relation between word frequency, word length, word co-occurrences, and text redundancies with reading times. For example, it could be expected that the effect of word frequency decrease with repeated reading, because readers do not need to rely on general frequency of occurrence any longer. Instead, optimization of the reading process due to repeated reading may capitalize on the idiosyncratic word frequency structure of the text. Similarly, the effect of word length could be reduced with repeated reading because a more superficial reading suffices to identify the now familiar words compared to when they were still unknown or unexpected. Of course, just the opposite might be that case as well. For example, simplified properties such as word length might become salient word markers when other word properties are already familiar to the readers because they have been reading the same text before. To our knowledge, the role of lexical variables in connected text reading has not been investigated yet.

Lastly, an examination of word co-occurrences is useful, because it can quantify sequential priming effects during reading, which is thought to play a major role in text reading [35]. Perhaps sequential priming effects gain in strength when the text is already known, as preceding words hold significant information for the reader about what is to come next. Sequential priming effects might also shed light on the distinction between word and phrase or sentence reading. Parafoveal information is not as present in the word unit condition, and readers might rely more on information present in the currently displayed word to facilitate reading of the following word [36]. In any case, effects of lexical variables hold potential information about the differences in reading performance we observed between text units, reader groups, or repeated readings.

### Word Frequency, Word Length and Co-occurrences

Word frequency and length are the two lexical variables most predictive lexical variables of reading speed and word recognition [37]. As we have speculated, especially word frequency could be informative about differences between reader groups or differences between first and repeated readings.

Global word frequencies were estimated from the USENET corpus [38]. To assess the effects of word frequency on word reading times, the logarithm of the word frequencies was correlated with each participant’s reading times for the word unit condition. To assess the effect of word frequencies on phrase and sentence unit reading times, word frequencies were summed into phrases and sentences and then divided by the number of words contained in each phrase and sentence. The phrase and sentence reading times were divided by their respective number of words.

To investigate the effects of word length, we compiled a vector of word lengths as the number of letters per word for ‘Arelis Complex.’ The procedure was the same as for word frequencies: The vector of word lengths was correlated with each participant’s word unit reading time series, and word lengths were averaged over phrases and sentences and correlated with the average word reading times for phrase and sentence units.

Similar to word frequency, lexical co-occurrences are tabulated over millions or billions [38], [39], [40] of words from multiple, distinct sources of text. We derived co-occurrence values using Latent Semantic Analysis (LSA; [41]) for all word pairs. The calculation of co-occurrence values was based on the online General Reading Corpus for 1^st^ Year College Students [42]. Similar to word frequency and word length, a vector of co-occurrence values was created for the story. However, this vector contained n-1 points, where n is the number of words in the story. As example, in the sentence “the boy ran across the old road”, the created time series would be the co-occurrence frequency of {the, boy}, {boy, ran}, {ran, down}, …, {old, road}. In theory, changes in the variability of this time series should index topical shifts of the story and the co-occurrences between adjacent words has successfully predicted semantic priming effects [39]. For phrase and sentence units, the co-occurrence values were again summed over the respective text units and divided by the number of words a phrase of sentence contained.

After preparation of the lexical variables and reading times, the resulting vectors of (average) word frequency, lengths and co-occurrences were correlated with each participant’s (average) word reading time. The correlation coefficients between reading times and lexical variables were then subjected to analysis of variance with the factors reader group and number of readings.

For word frequency, the correlation with reading times was stronger for phrase and sentence units compared to word units (*F*(1, 84) = 13.10, *p*<.001), but there were no significant effects of reader group or repeated reading, and no interactions between the factors (all *F*<1.88). Table 1 gives an overview over the reliability and average correlation strength for each text unit.

**Table 1:** Strength and reliability of the correlations between word frequency and reading times for word, phrase, and sentence units.

Text unit	Correlation	Intercept
word	*r* = −.022	*F*(1, 28) = 68.22, *p*<.001
phrase	*r* = −.047	*F*(1, 28) = 138.33, *p*<.001
sentence	*r* = −.048	*F*(1, 28) = 83.08, *p*<.001

For word length, the correlation with reading times was stronger for phrase and sentence units compared to word units (*F*(1, 84) = 104.70, *p*<.001). There were no significant effects of reader group or repeated reading, and no interactions between the factors (all *F*<2.43). Table 2 gives an overview over the reliability and average correlation strength for each text unit.

**Table 2:** Strength and reliability of the correlations between word length and reading times for word, phrase, and sentence units.

Text unit	Correlation	Intercept
word	*r* = .021	*F*(1, 28) = 56.45, *p*<.001
phrase	*r* = .117	*F*(1, 28) = 288.58, *p*<.001
sentence	*r* = .150	*F*(1, 28) = 311.04, *p*<.001

For co-occurrences, we observed to differences in correlation strength with reading times as a function of effect of text unit, reader group, or repeated reading (all *F*<1.88) with the exception of sentence units, where the negative correlation between co-occurrences and word reading times increased with repeated reading from *r* = −.003 to *r* = −.033 (*F*(1, 28) = 11.71, *p*<.01) – see Table 3 for a summary of the overall effects.

**Table 3:** Strength and reliability of the correlations between word co-occurrences and reading times for word, phrase, and sentence units.

Text unit	Correlation	Intercept
word	*r* = −.019	*F*(1, 28) = 50.34, *p*<.001
phrase	*r* = −.029	*F*(1, 28) = 35.17, *p*<.001
sentence	*r* = −.018	*F*(1, 28) = 16.62, *p*<.001

### Information Obtained from Word Frequency, Word Length, and Co-occurrences

The analysis of word frequency, word length, and sequential semantic priming effects through co-occurrences yielded only limited insight into the differences between the reader groups or the effect of repeated of reading. We did observe that the impact of co-occurrences increases with repeated reading in the sentence unit condition, where co-occurrences did not contribute to the performance for the first reading (mean *r* = −.003), but did lead to slightly faster response times in repeated reading (mean *r* = −.033). The sentence unit condition might have offered more salient features for the first-time reader compared to word and phrase unit reading. Upon re-reading, co-occurrences might have offered additional information that facilitated the ease of reading,. The question remains why no such effect was observed in the phrase unit condition.

Furthermore, phrase and sentence unit reading seems to generally bear stronger relations with lexical variables compared to word unit reading, which is expected if participants reading performance is confounded with their ability to reveal new words, yielding less accurate estimates of actual reading times.

More interestingly, however, is the general disparity between the reliability of the contributions of lexical variables and co-occurrences, and the amount of variance they explain in the case of self-paced text reading: Even in the best case these variables explain little more than 2% of the variance. This is in stark contrast to the majority of other studies using mainly single word or single sentence presentation, where the amount of variance explained is ranges from 15% to 35% [43], [44].

**Figure 6:**
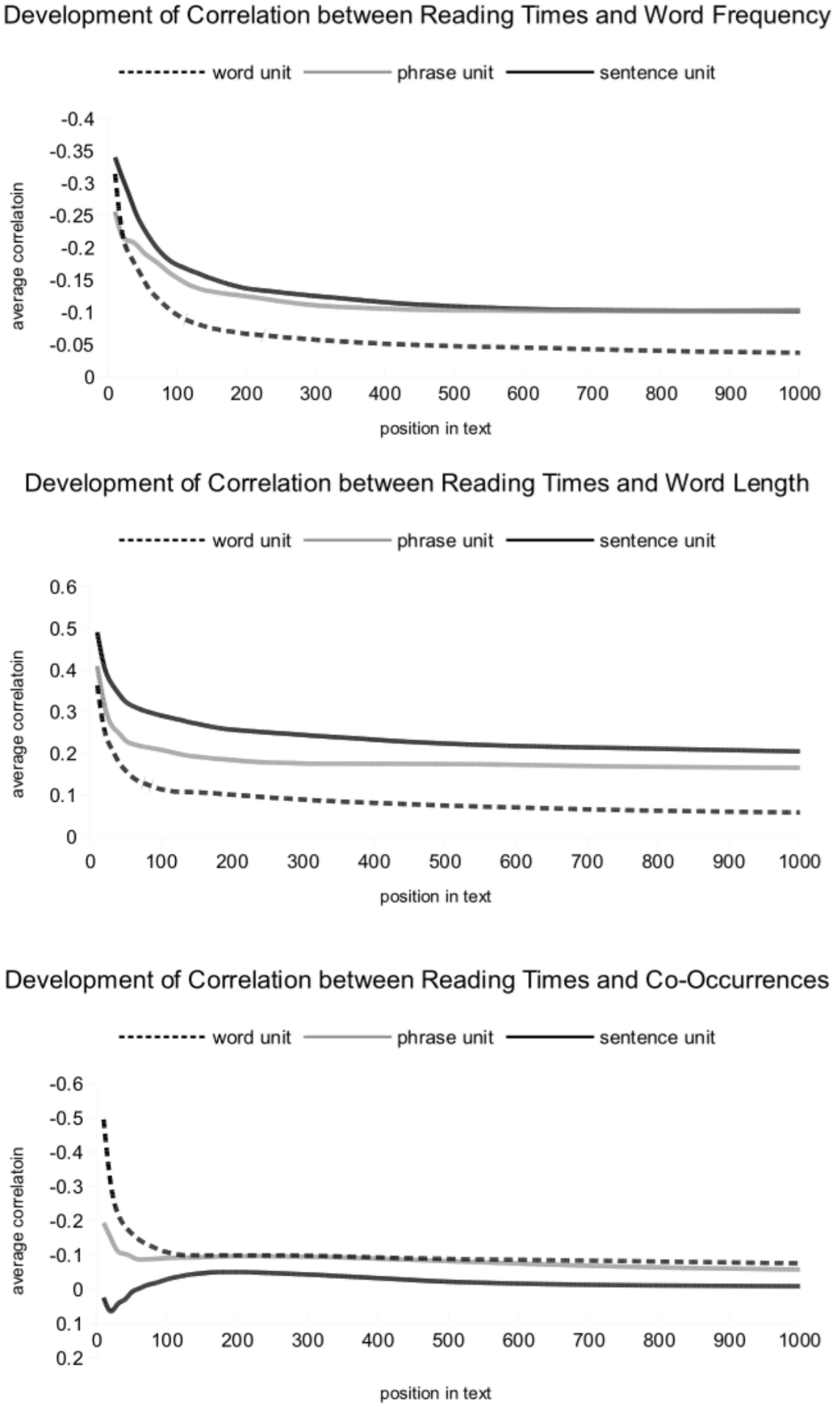
Average correlation between word frequency (top panel), word length (middle panel), and co-occurrences (lower panel) with reading times. The correlation is plotted as a function of the number of text units (words, phrases and sentences) considered, starting with the first 10 text units. As can be seen, the correlation strength is relatively high for the very first bins for each text unit, but dropping off as more and more words/phrases/sentences are considered.

Figure 6 displays the average correlation strength between word frequency, word length, co-occurrences and reading times for each text unit as sample size is increased, starting with the first 10 text units of each condition. The first bins show expected correlation strength as reported in other studies. However, correlations approach zero as sample size is increased (except for the correlation between co-occurrences and sentence reading times).

The relation between lexical variables and reading times seems to be greatly dependent on the presence of absence of the amount of prior information. When readers are new to the text, lexical features are important for the organization of the reading process (as marked by the high, initial correlation strengths). However, they seem to loose power as the text starts to provide its own context for the reader through its own idiosyncratic structure, which takes over all effects otherwise derived from general lexical measures. This would also explain why these effects remain stable under conditions of single-word or single-sentence presentation in other studies, where no larger, overarching constraints are build up throughout a given trial.

Henceforth, it seems warranted to test whether a lexical variable that is tailored to the individual text will yield stronger relations to reading times compared to a general descriptor such as word frequency. In the following sections we will first introduce Recurrence Quantification Analysis and then use this technique to build a recurrence portrait of the ‘Arelis Complex’ short story as described by Orsucci and colleagues [45] for the analysis of the text structure of poems. This recurrence portrait can then be reduced to a single vector that contains the idiosyncratic text redundancies inherent in ‘Arelis Complex’, which can be used as a predictor for word, phrase, or sentence reading times.

### Redundancies in Text Structure and their Uncovering by means of Recurrence Quantification Analysis

In order to make the outcome of the recurrence analysis of the short story–and how it leads to information about redundant text structures–more understandable, we will give a brief introduction to Recurrence Quantification Analysis (hereafter RQA) first. Then we apply it to ‘Arelis Complex’ and investigate the effects of the idiosyncratic text structure on reading times.

RQA has been used to analyze the behavior of physical and biological systems [48], [49], but has also applications in the social sciences. For example, RQA has been applied to identify the transition between problem solving strategies [50], reading behavior [18], [19], [51], force output control [52], motor control in athletes [53] or to gauge the quality of social relations between people [54].

RQA is a method to quantify the amount of recurrent structure in a sequence of data. The differences in the amount of recurrences in a data series is a powerful metric of its simplicity or complexity, and the different way in which a data series can be recurrent is highly informative [46]. Data that follow a regular pattern of variation repeat the same behavior often, producing a lot of recurrences and a simple regular recurrence structure. In more complex data series, recurrences become less frequent and the pattern of recurrences itself can change as well. RQA quantifies these aspects of recurrence and results in measures that can distinguish among different data sets.

The first step of RQA is the reconstruction of the so-called *phase-space portrait*. The phase space portrait can be inferred from any 1-D data series, (such as reading times) and is reconstructed as follows: A vector of the ordered data series is first plotted against itself. This can be done with a certain *delay*, a constant shift in one of the data series, which yield the higher-dimensional portrait of the system dynamics.

Second, the higher-dimensional phase space is used to define structure in a 2-D *recurrence matrix*. In a recurrence matrix, locations in the phase space that equivalent for each of the two data series (the original one, and the delayed one) are marked. The mathematical rationale and proofs can be found in [55]. The recurrence matrix is formed by following the order in which the data points were collected on both axes of a matrix, and then marking each point in the matrix at which equal values recur at the same location, along a shared trajectory, in the higher-dimension phase space. The recurrent data points are thus located in the neighborhood of equivalent values along a shared trajectory of the space that was formed using the delayed data.

**Figure 7:**
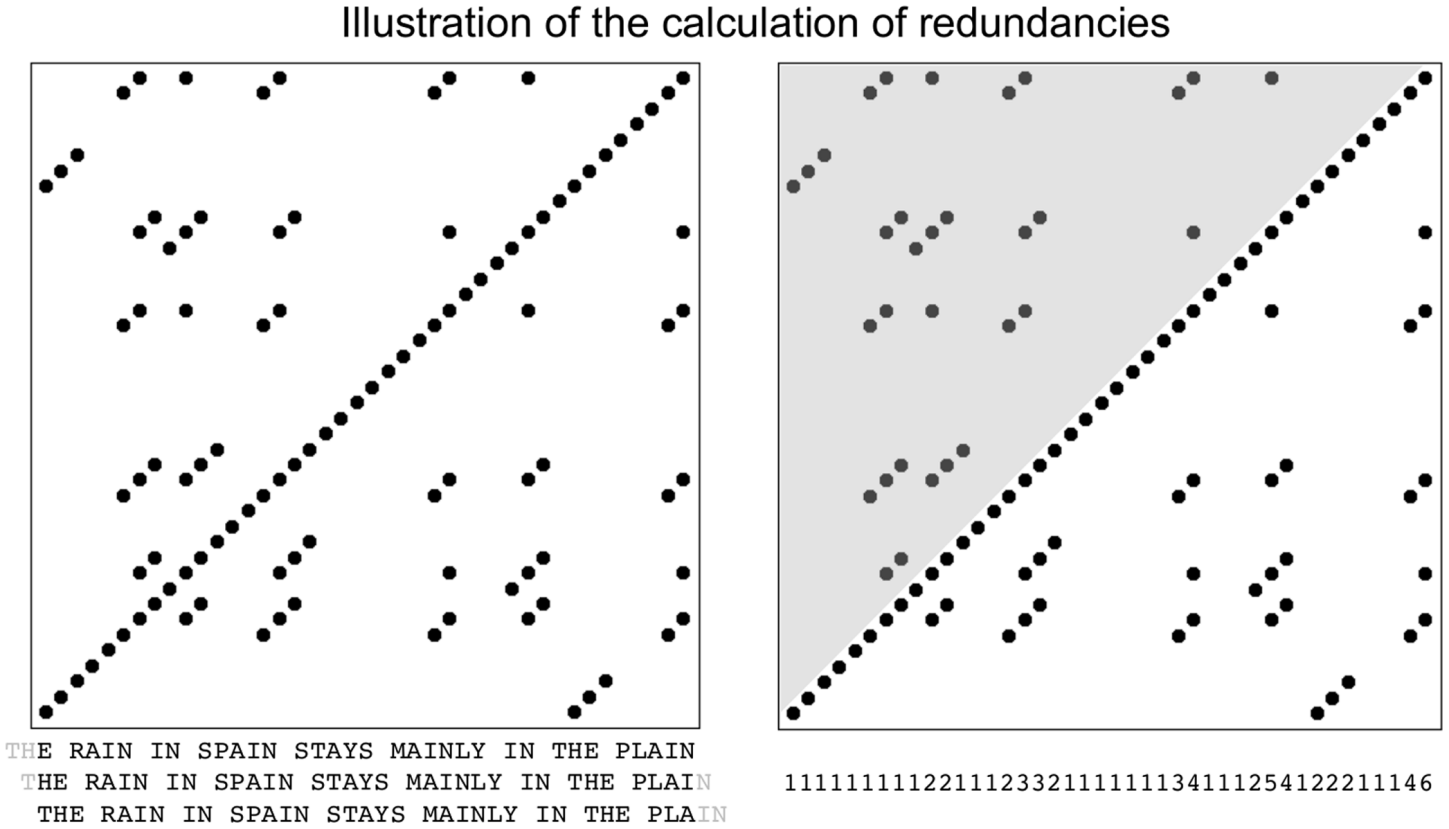
Recurrence analysis of ‘the rain in Spain stays mainly in the plain’. Left: Recurrence structure obtained from ‘the rain in Spain’ with delay of 1 and dimensionality of 3 (i.e., the text vector is shifted by 1 symbol 2 times and identical recurring trigrams are plotted as dots in the recurrence plot. Right: The recurrence structure of ‘the rain in Spain stays mainly in the plain’ is reduced to a single vector of redundancies, where the number of dots in each column are summed up for the lower-right half of the plot (the plot is symmetrical above and below the diagonal).

To illustrate the process for text data, we simply plot the sequence of letters and symbols against itself at delay 1 and delay 2, as illustrated in left panel of Figure 7, where the letter sequence of ‘the rain in Spain stays mainly in the plain’ is shifted by one letter two times. This procedure is used, in turn, to generate the 2-D recurrence matrix, in which identical trigrams of symbols are marked with a dot.

The recurrence matrix is formed by following the order in which the data points were collected and then marking each point in the matrix at which identical trigrams of letters recur, yielding the recurrence portrait displayed in Figure 7. The simple recurrence plot always has a diagonal line of points, which means that the data series is always identical to itself at delay 0. As we move away from the diagonal, we enter the space where identical structure in the letter sequence might potentially repeat itself at a later time. For example, the word ‘rain’ contains the trigram ‘ain,’ which we also find in ‘spain,’ and which will be identified in the recurrence matrix as a point off the diagonal.

To condense the recurrence matrix to a 1-D vector of redundancies, we simply sum up the number of points in each column of the matrix, as illustrated in Figure 7, right panel. Since the two halves of the recurrence plot above and below the diagonal are symmetric, we only sum over the lower of the two halves (the white one in the lower right). This vector can now be summed into word, phrase, and sentence units and gives an estimator of the overall lexical redundancy contained in these units as a text unfolds. This will give us a lexical metric that is tailored to the individual text structure, and not drawn from general text statistics. Perhaps an individual metric will yield greater correlation with the reading time performance, or will distinguish more sensitively between the different reader groups and repeated reading.

For ‘Arelis Complex’, we likewise chose a delay of 1 and embedded it in three-dimensional phase space (i.e., plotting the sequence against itself three times, each of them one step apart from the next), following recommendations of [45], who used RQA to examine the quality and complexity of poetry, to maximize the sensitivity of the analysis. The resulting vector was then summed into words, phrases, and sentences accordingly and correlated with the respective reading times, just as with word frequency, word length, and co-occurrences.

We observed that the overall correlations strength between reading times and text redundancies is less for phrases compared to word or sentence units (*F*(2, 84) = 3.17, *p*<.05). No other effects were apparent (all *F*<1.09). Table 4 gives a summary of the overall reliability and amount of explained variance of redundancies.

**Table 4:** Strength and reliability of the correlations between text redundancies and reading times for word, phrase, and sentence units.

Text unit	Correlation; *r^2^*	Intercept
word	*r* = −.097; *r^2^* = .009	*F*(1, 28) = 33.35, *p*<.001
phrase	*r* = −.056; *r^2^* = .003	*F*(1, 28) = 12.98, *p*<.001
sentence	*r* = −.116; *r^2^* = .013	*F*(1, 28) = 37.94, *p*<.001

We also conducted an analysis of redundancies by constructing a running count of words for the text. The overall results were the same, but the metric was problematic for the beginning of the text, as most of the words appeard only once, and hence there was not enough variation to calculate the correlations between frequency and reading times.

### Information Obtained from Text Redundancies

Contrary to our speculations, redundancies tailored to the individual structure of the text do not explain a greater share of the variance than general lexical variables. Overall, text redundancies are less correlated with reading time performance in the phrase unit condition compared to word-by-word and sentence-by-sentence presentation. That means that the idiosyncratic history of the text has even less influence in the case of phrase unit reading. We will come back to the possible meaning of this finding in the general discussion, after we have obtained more information about the dynamics of the reading times through recurrence and fractal analysis in the following sections.

In any case, the overall low contribution of the general lexical variables is arguably not simply a function of tailoring lexical statistics to an individual text, as individual text redundancies do not yield stronger or more sensitive relationships. Furthermore, the relation between text descriptors and reading performance has not been very informative about the nature of the differences between good and excellent readers, nor about the nature of repeated reading.

We obtained no effects for the word and phrase unit condition. There is a significant decrease of the facilitation effect of co-occurrence in sentence reading after the text had been read for the first time. This might be due to a better utilization of information in the sentence unit condition with repeated reading. However, the differences between the reader groups, as apparent in the central tendencies of reading times, are not picked up by changes in the relations between lexical variables and reading times.

Another alternative to the analysis of central tendencies and dispersion properties, as well as relation between reading times and lexical variables is an investigation of the time-dependent dynamics of reading times and their differences between reader groups or number of readings. In the next section, we present the results of RQA and Fractal Analysis of reading times that are capable of quantifying the structure of the evolution of the reading process in self-paced reading and generate further information about our reading tasks.

While RQA, which we already employed to identify idiosyncratic text redundancies, is capable of describing a variety of structural dynamical changes in time series [46], Fractal Analysis describes changes in fluctuation and self-affinity of a times series [47] – both analysis have already proven useful for cognitive and psycholinguistic investigations [18], [51].

### Recurrence Quantification Analysis of Reading Times

RQA cannot only be used to quantify nominal data, as we have illustrated with the text data above, but also continuous data, such as reading time. Similarly, it quantifies different aspects of the stability and predictability of a time series. RQA yields multiple quantitative outcomes variables, and more are currently under development. Not all of the variables are always meaningful for each data set, and often some of them will result only in redundant information [56]. In our analysis, we report two variables; percent recurrence (%REC), and percent determinism (%DET), and percent laminarity (%LAM). These three variables are percentages and their computation is not impacted by the size of the data sets, which makes them especially useful for comparisons between the different text units,. Second, there are significant differences in these measures between tasks, and the information they pick out of the reading times are redundant with other RQA output variables.

**Figure 8:**
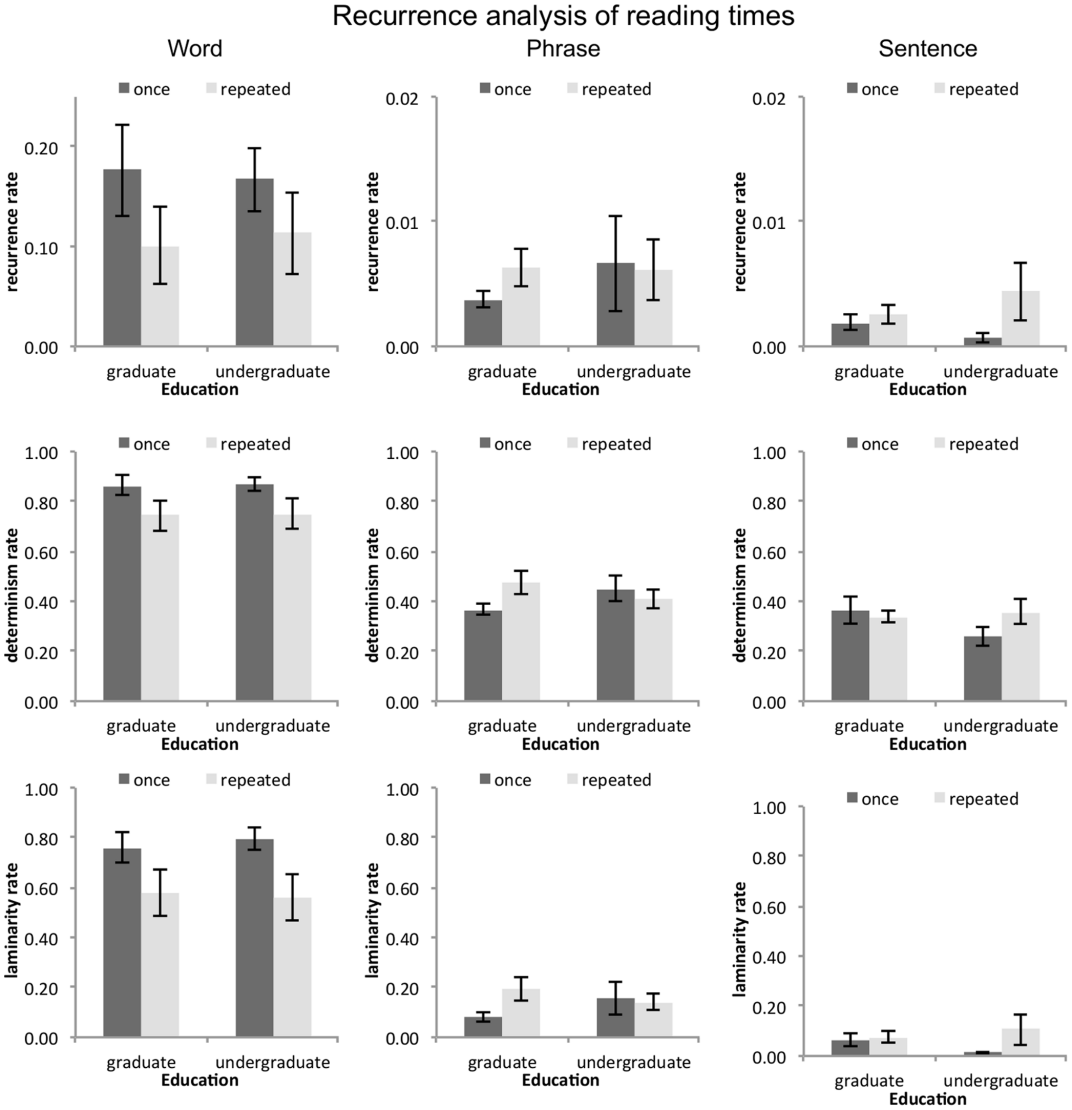
Results of recurrence analysis for words (left column), phrases (center column), and sentences (right column). The top row displays the values for %REC, the middle row displays the values for %DET, and the bottom row displays the values for %LAM. %REC and %LAM dropped with repeated reading in the word-unit condition, while no effects were apparent in the phrase and sentence reading conditions. However, %REC, %DET, and %LAM were lower for phrase and sentence-unit reading compared to word-unit reading.

Higher %REC (for the same value of the radius parameter) would indicate that the behavior is less spread out in the phase space, perhaps less perturbed. %DET equals the number of diagonally adjacent points in the recurrence plot divided by the total number of recurrent points, which estimates the degree of order in the data. For example, the extent to which readers fall into coherent trajectories of similar reading times across text units. High %LAM would indicate that there are only few overall changes in the dynamic of the responses over time, and that the performance is confined to few difference states. Low %LAM on the other hand would indicate that changes over time are erratic. Figure 8 shows the results for %REC, %DET, and %LAM. To uncover differences in the temporal structure of reading times produced by the different types of presentation, we will begin our analysis of reading time dynamics with an analysis of variance that includes the factor text unit.

Bigger text units – phrases and sentences – result in less %REC (*F*(2, 84) = 46.76, *p*<.001) compared to word units. No other effects were apparent (all *F*<2.81).

%DET in word unit reading is much higher compared to phrase and sentence unit reading (*F*(2, 84) = 131.31, *p*<.001). We also observed an interaction between text unit and repeated reading (*F*(2, 84) = 4.09, *p*<.05). To investigate this interaction, we broke down the analysis by text unit: For the word unit condition, we observed a drop in %DET with repeated reading (*F*(1, 28) = 6.13, *p*<.05).

Finally, %LAM indicates that there was more laminar structure in word reading times compared to phrase or sentence unit reading time (*F*(2, 84) = 148.74, *p*<.001). As we also observed an interaction effect of text unit and repeated reading (*F*(2, 84) = 7.60, *p*<.001), we broke down the analysis by text unit: Just as %DET, %LAM dropped with repeated reading in the word unit condition (*F*(1, 28) = 7.79, *p*<.05).

No other effects were apparent (all *F*<3.60).

### Information Obtained from Recurrence Quantification Analysis of Reading Times

The results obtained from RQA of reading times highlight differences between the text units, especially between the word unit condition compared to the phrase and sentence unit conditions. Higher %REC values for the word unit condition mean that word unit reading is much less variable compared to phrase or sentence unit conditions. The performance is confined to much more narrow pockets of the phase space, and is overall more alike. %DET and %LAM further specify these differences: Higher values for %DET mean that individual states in phase space are more often similar for word unit performance, and also that trajectories (i.e., change in states in phrase space) recur more frequently. The higher values for %LAM show that performance in general is much more stable for word units compared to phrase or sentence units, and that the dynamics do not, or only slowly change.

These findings make sense in several ways: First, overall variability in word unit responses is smaller, and thus, performance will be relatively more homogenous than for bigger text units. Second, word unit performance takes place on a subscale of phrase and sentence unit performance: Changes from one sentence to another, for example, are actually brought about by changes in sets of words. Hence, the changes on the word scale will look insignificant from the perspective of the scale of sentences. They are simply a stable state by which a sentence is made up.

Furthermore, we found statistically significant decreases in %DET and %LAM with repeated reading in the word unit condition. This pattern of effects in the correlations with reading times was expected, since it indicates some sort of loss of structure in the participants’ performance with repeated readings. Less %DET means that there are fewer repetitions in the sequences for key-presses, and fewer %LAM means that performance becomes more perturbed. So for the word unit condition, the text had a stabilizing influence on participants’ performance, which seem to have waned with repeated reading. Specifics of the text – such as a certain passages of terms – loose their importance for text understanding, as they are now already known to the reader. The drop in %DET and %LAM might reflect just that.

Differences between reader groups are not revealed by RQA and no differences are revealed for the bigger text units. A question of interest is, whether these differences do not exist (at least in self-paced measures of reading) or whether text reading is such an idiosyncratic process that no common structure is easily found between readers and manipulations. If differences do not exist, performance is, at its core, very much alike across conditions. Otherwise, idiosyncrasies play out as higher statistical uncertainties. The wide range of overall story reading times, varying from 17 to 90 minutes, but all resulting in good text comprehension, points to the high idiosyncrasy in text reading performance.

This hypothesis can be tested using a variation of RQA, Cross Recurrence Quantification Analysis (hereafter CRQA). CRQA reveals commonalities among readers in a group that are due to the common accommodation of task demands, including how the text-unit conditions affect readers similarly or differently. By employing CRQA, we build our analysis only upon the structure that different readers have in common, instead of the whole structure contained within their performance, including all of the readers’ idiosyncrasies. It furthermore allows to test the hypothesis that repeated reading goes along with a kind of destabilization of performance in the word condition. This should be an effect due to commonalities in performance that are text-induced.

### Cross Recurrence Quantification Analysis

CRQA extends recurrence analysis in much the same way that a correlation between two random variables extends auto-correlation of a single random variable. In the latter case, we evaluate a data series against itself, and in the former case against another data set. In CRQA can be thought of as a kind of nonlinear correlation analysis developed to test whether, and the extent to which, the dynamics of two systems evolve in common [57]. If they do, then common sources of constraints, such a reading the same text, are implicated, where a change in constraints is associated with a change in the degrees of freedom of trajectories in the state space. More tightly constrained dynamics have fewer degrees of freedom and less tightly constrained dynamics have more degrees of freedom.

The procedure for conducting CRQA is nearly the same as that for conducting RQA, the primary difference being that two data sets are contrasted instead of only one [58]. We used CRQA to compare the shared dynamical structure among readers who read the same text under the same conditions. So, for instance, we ask whether two undergraduates produced shared patterns of reading times because they read the same text, advancing it identically, unit by unit in spacebar presses. The idea behind this analysis is that readers may possibly entrain to a text in self-paced reading or adopt the same strategy for advancing the text. Such commonalities may yield parallel dynamics that can be captured by CRQA.

**Figure 9:**
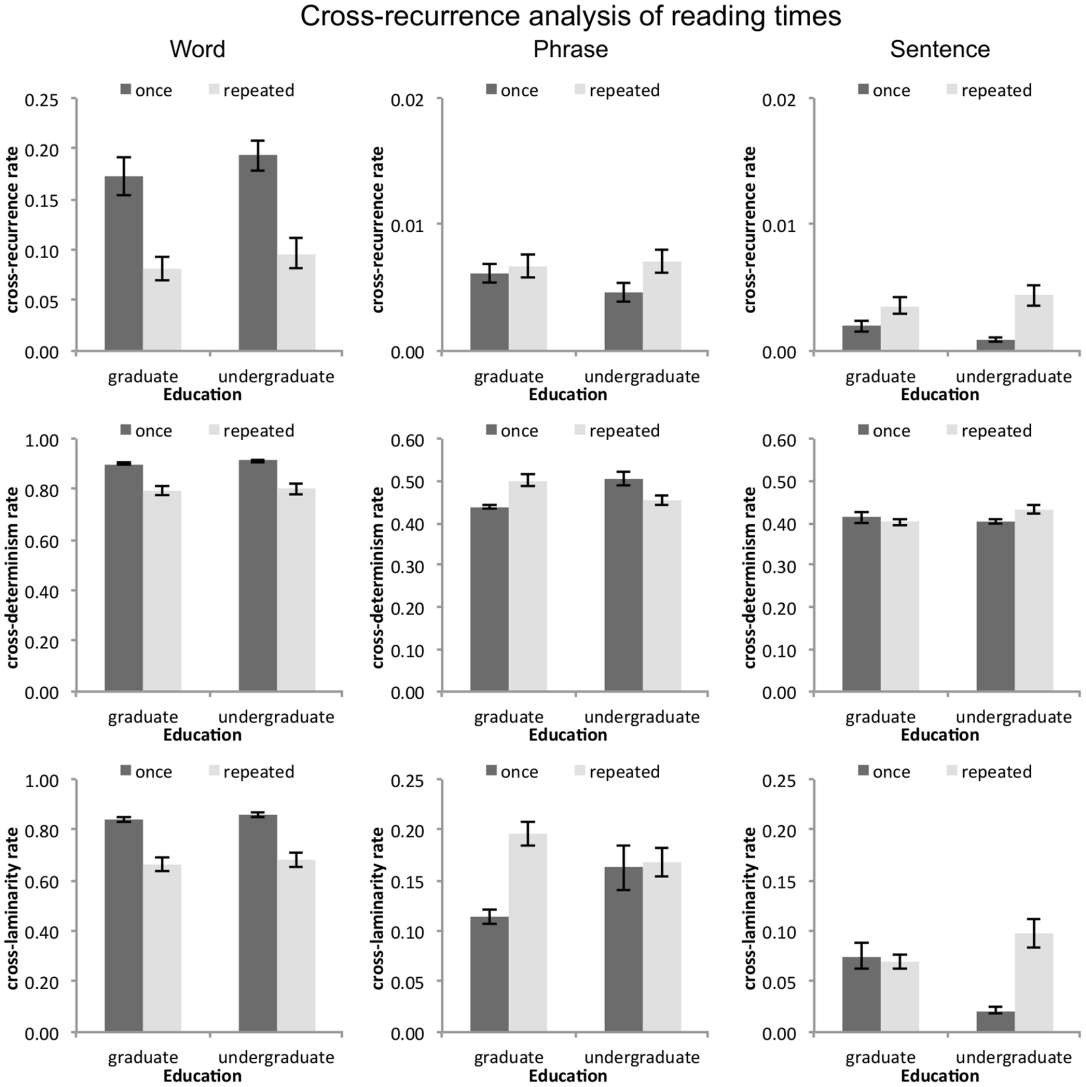
Results of cross recurrence analysis for words (left column), phrases (center column), and sentences (right column). The top row displays the values for shared %REC, the middle row displays the values for shared %DET, and the bottom row displays the values for shared %LAM. The three text-unit conditions differ in the effects on the recurrent measures: In the word-unit condition, shared %REC, %DET, and %LAM decrease with repeated reading for both reader groups. In the phrase unit condition, shared %DET and %LAM increase with repeated reading for graduate readers, but decrease (or do not change) for undergraduate readers with repeated reading. In the sentence-unit condition, shared %REC increases for both groups with repeated reading, while shared %DET and %LAM only increases for undergraduate readers with repeated reading.

Each condition included eight readers and we examined all possible pairings, which yielded 28 possible pairings in each cell of identical reading conditions. The data were transformed into z-scores prior to the analysis. For the parameter settings for the CRQA analysis, we used the averaged individual parameters for any pair of data sets. Figure 9 gives an overview over the results for shared %REC, %DET, and %LAM. When the effects of repeated reading and reader group on the CRQA variables are assessed, we obtain the following picture:

Shared %REC decreased with repeated reading in the word unit condition (*F*(1, 108) = 33.17, *p*<.001), but increased with repeated reading in the sentence unit condition (*F*(1, 108), = 20.05, *p*<.001).

Shared %DET decreased with repeated reading in the word unit condition (*F*(1, 108) = 55.27, *p*<.001). For the phrase unit condition, there was an interaction between reader group and repeated reading on shared %DET (*F*(1, 28) = 21.63, *p*<.001), indicating that shared %DET increased for graduate readers, but decreased for undergraduate readers. For the sentence unit condition, we observed an interaction between reader group and repeated reading on shared %DET (*F*(1, 28) = 4.54, *p*<.05), indicating that shared %DET decreased for graduate readers, but increased for undergraduate readers.

Shared %LAM decreased with repeated reading in the word unit condition (*F*(1, 108) = 64.12, *p*<.001). For the phrase unit condition, there was a main effect of repeated reading on shared %LAM (*F*(1, 108) = 8.51, *p*<.01) which was qualified by an interaction between reader group and repeated reading (*F*(1, 108) = 6.79, *p*<.01), indicating that shared %LAM increased for graduate readers with repeated reading, but not for undergraduate readers. For the sentence unit condition, we observed a main effect of repeated reading on shared %LAM (*F*(1, 108) = 11.75, *p*<.001) which was again qualified by an interaction between reader group and repeated reading (*F*(1, 108) = 15.86, *p*<.001), indicating that shared %LAM increased for undergraduate readers with repeated reading, but not for graduate readers.

No other effects were apparent (all *F*<3.10).

### Information Obtained from CRQA of Reading Times

Cross Recurrence Analysis of paired data sets corroborates the results obtained from RQA of individual data sets: The word unit condition stands out against the phrase and sentence unit condition in that its overall values for %REC, %DET, and %LAM suggest much more stable dynamics. Furthermore, we observe strong effects of repeated reading. Wallot and Van Orden [18] interpreted the drop in recurrence measures with repeated reading as a sort of individuation of performance, where common constraints of the text exert less influence on readers’ performance. Of course, the results could suggest an overall loss of structure with repeated reading and overall more erratic performance. However, we also observed a decrease in the distributional tails of reading times and it rather seems that even though participants’ performances evolve more differently in time after the first reading, they are also more constrained within each individual.

Overall, the word unit condition seems to stabilize the performance of readers, not only in terms of the observed dynamics, but also in that it yields simple, but clear, main effects of repeated reading. The bigger text units, on the other hand, give rise to observed differences between reader groups, which are observed for phrase or sentence reading. We will discuss the results observed for sentence reading first.

For sentence unit reading, %REC increased with repeated reading for both reader groups. This is in contrast with the word unit condition, where shared %REC decreases with repeated reading. Maybe then, overall, repeated reading plays out in an increase of constraints of sentence units on reading performance. Sentences guide reading much more for the second reading, while they perturbed reading performance the first time. This would be in line with the observed decrease in standard deviation and steepness of distributional tails, which indicate that performance is less dispersed overall. We also observed a statistically significant increase in the role that co-occurrences play for sentence unit reading performance with repeated reading, suggesting that the performance is tied a little closer to some of the text’s properties.

Furthermore, sentence unit reading now reveals clear differences between graduate and undergraduate readers. Performance dynamics are either stable for graduate readers across repeated readings, while they change for undergraduate readers across repeated readings (%DET and %LAM), or their change is disproportionally greater for undergraduates (the marginal interaction effect observed for %REC). These outcomes could be expected if graduate students’ reading performance is always close to a performance ceiling, even when reading a story, sentence-by-sentence, for the first time. That is, graduate students in English literature are über-fluent, compared to fluent undergraduate readers [18].

The change for undergraduate students always indicates an increase in orderliness and structure of the time course of reading (higher %DET and %LAM with repeated readings). This corroborates the hypothesis that an increase in reading fluency for sentence unit reading might go along with a tighter coupling of text structure and performance. The observed general increase in %REC leads into the same direction. There might be commonalities between an increase in reading fluency due to the reader’s general reading experience (as perhaps captured by the differences between graduate and undergraduate students) and an increase in reading fluency due to specific item or text knowledge (as captured by repeated reading of the same items or texts [21]).

The picture we get from the shared dynamics of phrase unit reading is a little less clear. Overall, the outcomes for the %DET and %LAM measures, present a kind of mirror image of what was observed in the case of sentence unit reading. Now, graduate students show an increase in %DET and %LAM, while undergraduates show no change (%LAM) or a decrease (%DET) with repeated readings. One could speculate that graduate students’ repeated reading of phrase units is more like sentence unit reading, where performance is now more guided by text properties, while the undergraduate readers’ strategy is to free themselves of the constraints of the text, revealing phrases more flexibly, as observed in the word unit condition. However, such changes are not backed up by observations of changes in the relation to lexical variables (the only change we observed was a general drop of the correlation between phrase reading times and redundancies with repeated reading), nor is the patterns as clear cut. Also, while phrase units are obviously multiple-word units, they are not simply a form of smaller sentence units, which might explain the somewhat peculiar results observed in this reading condition, as we will discuss later on.

To conclude our analysis of self-paced text reading, we introduce one last analysis of reading dynamics that capitalizes on systematic changes of variance in the reading data over time. Fractal analysis captures the change of magnitude and frequency of fluctuations, and thus gives us information about the reading process, which can be related to general considerations about voluntary cognitive performance [59], [60].

### Monofractal Analysis

Monofractal analysis of reading times quantifies scaling relations between fluctuations in the frequency and magnitude of reading times. Scaling relations imply that long-range correlations exist in the data and that either the variance of the data series is nonstationary or both the mean and the variance are nonstationary. Nonstationary descriptive statistics cannot be trusted to be stable estimates of population parameters [34].

In such a case, monofractal analysis estimates a scaling exponent alpha, which quantifies the scaling relation and thus the nature of the long-range correlation, which holds information about the task demands or the participant’s skill [61].

We followed the guidelines of [45] in preparing the reading times for the spectral analysis. Although outlier reading times are in principle legitimate data values in nonlinear analyses, sufficiently extreme outliers can bias a spectral analysis, possibly prompting a false rejection of conventional analysis. Thus, extreme outliers were removed to ensure valid conclusions in that regard. Word reading times less than 100 ms or greater than 2,500 ms were eliminated from the spectral analysis, as well as sentence reading times less than 100 ms or greater than 20,000 ms. In the second step, all the reading times that fell outside of three standard deviations of a participant’s average reading time were removed, as well as linear and quadratic trends in the time series. Finally, the thus trimmed and detrended data were subjected to spectral analysis, using the Fast Fourier Transformation.

The spectral analysis of each participant’s reading times was plotted on log-log axes. The x-axis in the plot tracks how often changes of particular magnitudes occur, and the y-axis tracks the magnitudes of variation as changes in reading times. The relation between size of change and frequency of change is estimated by a least-square regression line, which quantifies the relation between size and frequency in the slope of the regression line. The end result estimates a scaling relation between power (*P*(*f*)) and frequency (*f*): *P*(*f*) = 1/*f’* ^α^.

**Figure 10:**
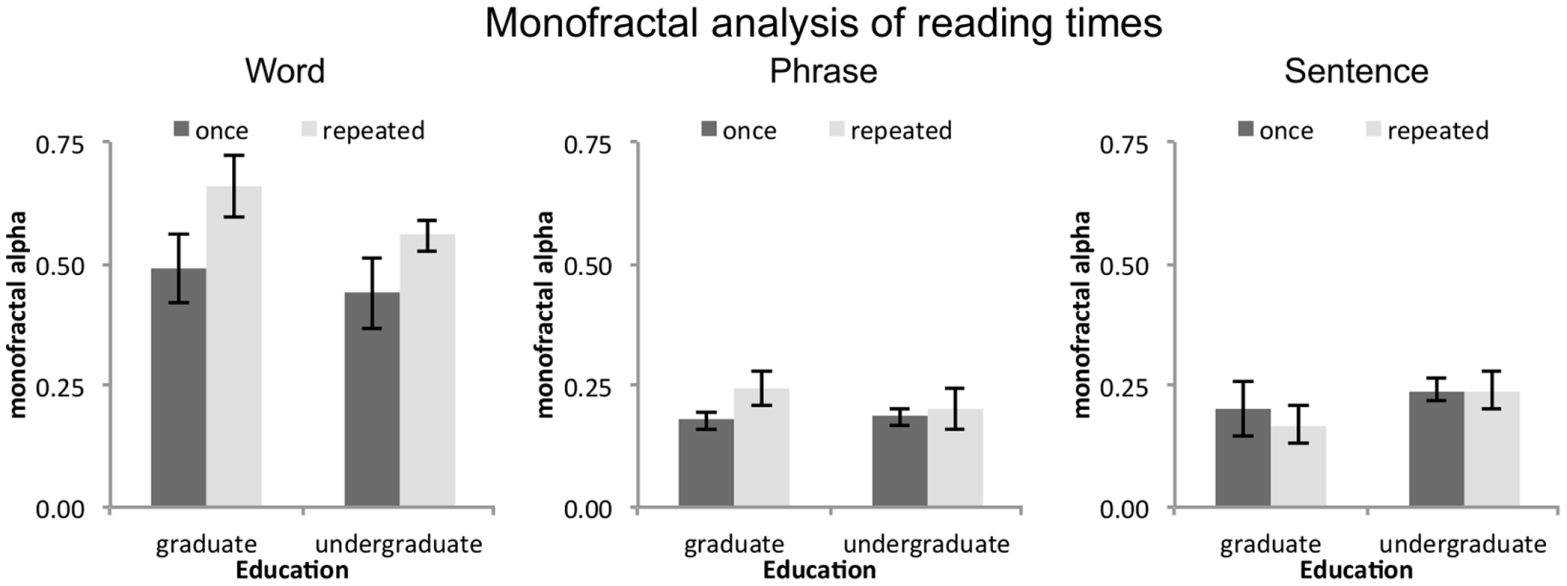
Monofractal α values for word (left), phrase (center), and sentence (right) reading times. Word reading times become increasingly long-range correlated with repeated reading, while no such change is observed for phrase and sentence reading times. While word reading times show generally a high degree of long-range correlation, phrase and sentence reading time fluctuations are much more locally determined.

Figure 10 portraits the results of the analysis. We observed a main effect of text unit (*F*(2, 84) = 66.72, *p*<.001), indicating that scaling exponents of word unit reading times are higher than those of phrase and sentence unit reading times. We also observed that scaling exponents increase with repeated reading (*F*(1, 84) = 4.28, *p*<.05). However, this effect was qualified by an interaction between text unit and repeated reading (*F*(2, 84) = 3.02, *p* = .054). To investigate this interaction, we broke down the analysis by text unit: α increased reliably with repeated readings in the word unit condition (*F*(1, 28) = 5.27, *p*<.05), but no other effects were apparent (all *F*<1.82).

### Information Obtained from Monofractal Analysis

The difference between word unit (mean α = .55) and phrases and sentence unit (both at about α = .19) presentation conditions can be interpreted as a difference in task demands. Task demands are sources of involuntary control [59], suggesting that phrase and sentence unit presentation increase the influence of task demands on performance measures.

However, the difference between the word condition on the one hand and the phrase and sentence conditions on the other hand is not exclusively in the linguistic or psychological properties of text units, but maybe also in the strategy that the reader brings to the task – it may be that the word unit condition also encourages different sources of control: As we have pointed out, words are read and understood almost immediately, and more quickly than the time it takes to initiate and follow through with pressing the space bar. Skilled silent undergraduate readers reach average speeds of around 300 words per minute [4] – close to simple reaction times, and faster than typical performance in a tapping task in which undergraduates produce repeated key presses at their self-selected comfort pace [62].

An efficient strategy in the word unit condition might be to find a fast pace for repeatedly pressing the space bar and simply read the words apace as they appear across the screen. This strategy explains the data nicely because the average α value in the word unit condition is within the range of values found in tapping performance [63], [64]. The hypothesis is also consistent with the changes due to number of readings of the story, where α increases with repeated reading, showing more tapping-like performance in terms of long range-dependencies, which go hand in hand with a decrease of text influence on the performance that we see in the recurrence and cross recurrence measures.

The latter result makes sense because graduate and undergraduate readers would not obviously differ in a tapping strategy, and the former result makes sense if this strategy is more reliably engaged in during repeated readings when both graduate and undergraduate readers are familiar with the story. For instance it is possible that less familiar words or complicated passages would increase word reading times intermittently but sufficiently in the first reading to supply unsystematic perturbations of measured values. A subsequent re-reading of the story could dampen these perturbations as repetition of story elements yields consequently faster comprehension of the now familiar words and sentences [18].

Together with the results from cross recurrence analysis, the results of fractal analysis show how the different text units stabilize reading performance in different ways: the word unit condition is in some sense more stable or constraining, since it levels differences between readers and produces clear and simple effects of re-reading. The overall performance is stabilized, which is also highlighted by the comparatively larger recurrence values. Fractal analysis reveals how individual responses – viewed as one-on-one mappings of a particular word and a particular response–are relatively unstable: The fractal dynamics suggest very strong carry-over effects, in fact, as we have laid out, very similar to tapping performance.

Phrase and sentence unit performance is much less structured overall, giving rise to very dispersed recurrence portraits and very low scaling exponents, indexing nearly (but not fully) uncorrelated trial-to-trial transitions. At the same time, the correlations between reading times and lexical characteristics of the text are somewhat higher for the bigger text units, and their cross recurrences increase with repeated reading, indicating a strengthening of the influence of text properties on performance.

### Multifractal Analysis

Monofractal analysis estimates a single fractal dimension across an entire data set and assumes that a data series has a stationary fractal dimension. This assumption is not always true however. Human response time data are sometimes monofractal, but more often the fractal dimension is non-stationary, changing during the course of data collection.

**Figure 11:**
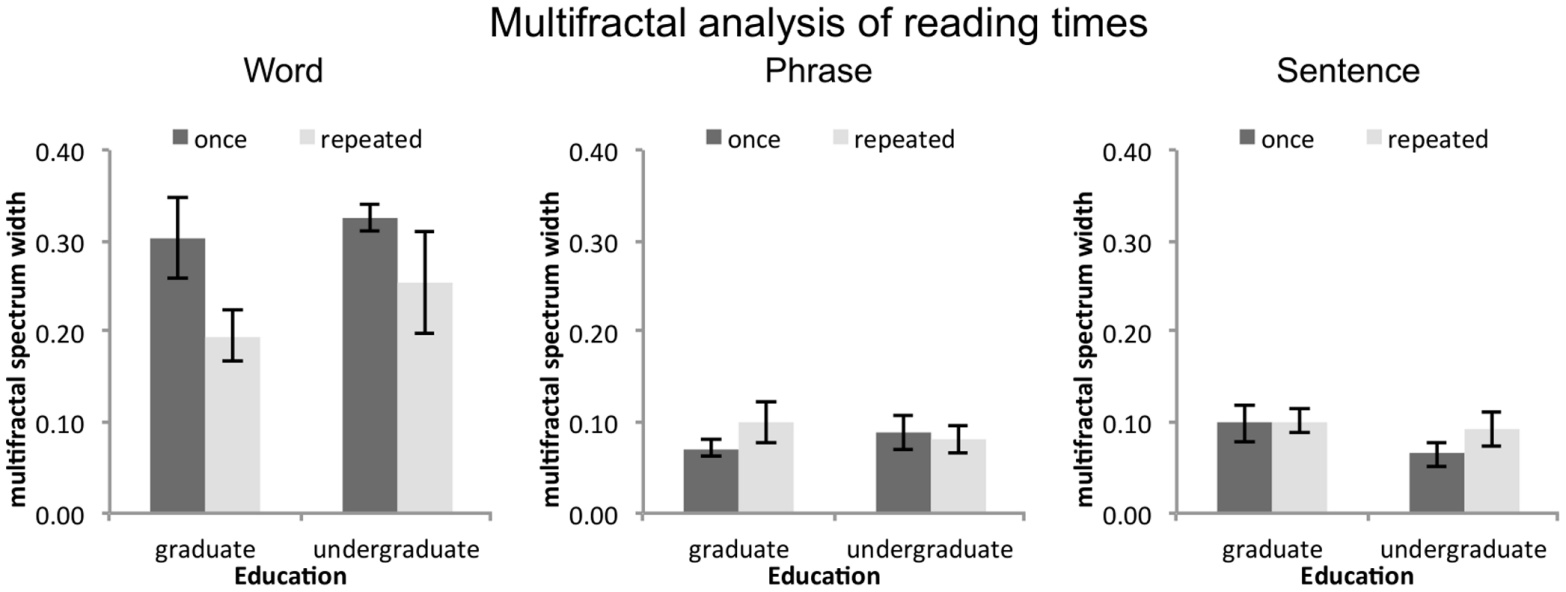
Multifractal spectrum width for word (left), phrase (center), and sentence (right) reading times. Multifractality decreases with repeated reading in the word-unit condition, while multifractality is absent in the phrase and sentence reading conditions.

Multifractal analysis tests whether a data set has a stationary fractal dimension and whether the dynamics of a performance are better described by multiple fractal dimensions, varying across a multifractal spectrum. It is an extension of monofractal analysis and assesses the heterogeneity of variation a data set. A positive outcome implies that the same data series entails multiple different organizations of the system that performs a task [65]. Figure 11 summarizes the results.

As with monofractal analysis, we observed differences in the multifractal spectrum between word unit reading and phrase and sentence unit reading (*F*(2, 84) = 38.82, *p*<.001). Also, we obtained an effect of repeated reading (*F*(1, 84) = 6.21, *p*<.05), which was qualified by an interaction between text unit and repeated reading (*F*(2, 84) = 4.30, *p*<.05): The multifractal spectrum contracted with repeated reading in the word unit condition (*F*(1, 28) = 5.78, *p*<.05). No other effects were apparent (all *F*<2.42).

### Information Obtained from Multifractal Analysis

The results of the multifractal analysis corroborate, but also expand, the results obtained from monofractal analysis. The word unit condition again stands out as being more variable and turbulent compared to the phrase and sentence unit conditions, and repeated reading affects the homogeneity of variability of word reading, but not of phrase or sentence reading.

Multifractal analysis also shows that phrase and sentence reading time are well characterized by the monofractal scaling exponent (which in turn is close to white noise), while this is not the case for word unit reading times, which are clearly multifractal. Multifractality is due to a special heterogeneity of variance observed when performance is governed by a multitude of scaling relations, which govern the dynamics of different scales and combine multiplicatively to yield the observed responses.

While monofractality increased with repeated reading, multifractality decreased. This might mark the transition from a more text impacted word-revealing strategy to a less impacted one: In the former, text units disrupt the strong long-range correlations, which result in slightly lower monofractal alpha values, but induce more pockets of strongly correlated performance, where the text exerts greater influence again. In the latter, we observe much more of a smooth execution of motor skill whose variation is of a different kind.

The presence of multifractality can be interpreted in terms of changes in coupling over time (in our case, over the course of the performance) and this might also be the key to understanding how lexical descriptors influence the reading times of word unit reading: Performance overall is only weakly related to word properties, resulting in monofractal exponents that are similar to those observed in tapping behavior. However, the text perturbs this tapping performance once in a while–for example during difficult text passages – where either reading times become erratic and/or temporarily (un)correlated with the variations in word properties. Moreover, the presence of substantial multifractal scaling in word reading times suggests that the perturbations are themselves long-range correlated. They depend upon the reading history and not only on the actual text read at that moment.

It can be speculated that the stabilizing effects of the bigger text units, phrases and sentences, would result in a smaller multifractal width and hence in a more stable relationship between response times and lexical variables. This could be a partial explanation for the overall somewhat stronger relation between lexical variables and reading times for phrases and sentences.

## General Discussion

After careful analysis of the self-paced text reading data and employing a variety of analysis methods, we have learned about 1. the nature of connected text reading, 2. the differences and commonalities in reading performance between the three different text unit, and 3. the role of reading fluency, as it shows itself in repeated reading and between reader groups who are both fluent readers, but supposedly differ in habitual reading fluency.

First, connected text reading tasks result in complex patterns of performance. Standard effects (such as the influence of word frequency on reading times) that have been found to be important for performance in single-word tasks, such as word naming or lexical decision, play only a minor role in complex reading. The comparatively small amount of variance explained by lexical variables, the long transient of average reading times at the beginning of text reading and the overall heterogeneous variance in performance suggest that text reading differs in important aspects from word or sentence reading. Text reading performance is somewhat better dissected by a quantification of the time course of reading performance, and the stability and complexity of reading performance seems to capture interesting aspects of text reading.

Second, the three text units used in the self-paced reading task (words, phrases, or sentences) impose different constraints on reading performance and yield patterns of reading times that refer jointly to task demands and readers. That is, although all three involve reading of the same text, they reflect reading differently in the reading time data they produce – so differently that they cannot be easily equated along a common quantitative dimension. Task demands in these three conditions are sufficiently different that they essentially create three different tasks.

Third, reading fluency is not just a simple consequence of text knowledge, as the effects of repeated reading are different from those of general reading experience. Also, reading skill develops well past the acquisition of literacy, as PhD candidates in English Literature show systematically different reading performance. While all participants displayed qualitatively different reading performances across the three text-unit presentation conditions, and between single and multiple readings of the story, performance of graduate students in phrase and sentence reading differs in development and stability from that of the undergraduates. Hence, it can be supposed that reading fluency, viewed as the pinnacle of reading ability, develops well past the acquisition of literacy and can be related to an increased stability in the dispersion and dynamics of reading performance.

We summarize the basis of these speculative conclusions in more detail in the following sections.

### Reading Text

We tried to trace the effects of local word features on reading times using the commonly used and well-researched lexical variables word length and frequency to predict changes in reading times. These two variables show robust effects in most laboratory reading tasks, but the amount of explained variance was minimal in our reading task. Similarly, when co-occurrences were used to estimate the effect of sequential semantic priming in text reading, we obtained a statistically significant effect, but again only of minor magnitude. Furthermore, when the idiosyncratic text structure was taken into account through lexical redundancies, this basic picture did not change. Hence, the overall weak relation between reading times and lexical variables is not simply a matter of fitting them to a particular text.

Nevertheless, it cannot be said that lexical variables play no role in text reading: when the development of the correlation between reading times and lexical variables is examined. For the very first few words, phrases, and sentences, we consistently observed a good correlation between lexical variables and reading times. It can be speculated that in the absence of contextual constraints, general lexical features play an important role. In reverse, this might imply that texts construct their own context as they increasingly provide information to the reader, which would also be consistent with the observed power-law decrease in reading times across all conditions. Hence, actual text reading performance is not well gauged by such measures beyond the point where a participant becomes familiar with the text’s content. Just as it has been shown that the primacy of literal over figurative meaning is lost in actual language use [66], it seems that primacy of individual word properties is lost in actual text reading.

Further evidence for the role of idiosyncrasies in text reading comes from the comparison of recurrence measures of reading times with cross-recurrence measures of reading times. Recurrence measures of reading times, are bracketed by relatively large standard errors compared to the cross-recurrence measures. Among other things, this means that the individual evolutions of reading performances are poorly captured by a central tendency *across* readers. Individual differences between readers are great, even though their performance eventually leads all of them to a good, and similarly accurate understanding of the story. On the other hand, cross-recurrences, dynamics that the readers share, reveal more narrowly dispersed structure (and also reveal differences between reader groups and repeated readings, which discuss in the following sections). Hence, there are similarities between participants’ performance due to reading the same text, but these are not consistent throughout the whole reading episode. They rather reveal themselves intermittently in the course of reading. Taken together, it seems that text reading is a very idiosyncratic process on the side of the reader, which is only poorly captured by properties of the text alone.

Of course, self-paced reading is also a laboratory task that is quite different from how people read when they hold a book or pad in their hands. Other methods have to be employed to corroborate these findings. Nevertheless, the picture of reading we see is more that of a production task (such as writing [67], [68]), and not so much a simple perception-response task. The product is the meaning that is constructed by the participant, entailing his or her personal knowledge, intention and interpretations, a process that is maybe much more obvious when reading is directly investigated as reading of texts instead of individual words and sentences.

### Reading Words, Phrases and Sentences

Reading performances of the three types of text units (words, phrases, and sentences) reveal qualitative differences in long-range correlational structure (i.e. fractal dimension), as well as dissociative changes in participants’ performance over reading units and multiple readings. Yet the ordinary goal of task contrasts is to find their common core. Presumably the common core would refer to reading itself, distinct from the idiosyncrasies of reading tasks–especially when, as in our case, the text remains the same. No reading process is shared identically across the three tasks, at least none is revealed in the data across the different analyses.

Conditions that advance the text in word units differ greatly from both phrase unit and sentence unit conditions. The fractal pattern across key press times is much stronger for word unit presentations. As we already suggested, the prominent fractal pattern is consistent with the idea that spacebar pressing takes longer, on average, than word reading times. The average reading time of roughly 250 ms in the word unit condition is not much longer than simple reaction times, for instance, when participants are dedicated exclusively to producing a rapid response. On this basis, we interpreted the key presses to be rate limiting: the key press primarily shapes performance across word reading trials, not the other way around. The likely effect on performance by text and word properties is to occasionally perturb the pattern of key pressing such that it departs toward slower reading times. This might happen when rare words or difficult passages of text come up. This ‘perturbation’ (i.e., longer, but also more variable reaction times) captures a complex, but systematic relation, as the results of multifractal analysis suggest, and is not simply some sort of error response that occurs once in a while.

Word unit reading reveals a pervasive effect of repeated reading, resulting in decreased shared recurrence, decreased multifractality, and a more shallow distributional tail, decreased %LAM, decreased %DET, and increased monofractal exponents. As we have already discussed, response times in the word unit condition likely reflect more the dynamics of the participants’ tapping, less actual word unit reading times. Repeated reading as it is reflected in word unit reading then minimized the intermittent effects of text passages on reading performance, resulting in fewer extreme values (shallower distributional slow) and fewer bursts in reading times (smaller multifractal spectrum, less shared %LAM). Usually, extreme values and heterogeneous variance are thought of as disruptions or instabilities of performance, but the simultaneous decrease in shared %REC and shared %DET indicates that they were capturing the effects of the text on the readers’ button press performance as well, having an ordering effect which is weakened by repeated readings, when the text is known to the reader. The increase in monofractal exponents toward 1/*f* noise, which is empirically much more often observed as going together with heavier distributional tails and higher multifractality, makes sense in terms of a qualitative change in performance, which has gone from a mixture of tapping and reading to mostly tapping, exhibiting stronger, but also simpler long-range correlations.

Conditions that advance the text in phrase units differ from sentence unit conditions, as well. Regarding the absolute values of the measurements, the phrase unit condition seems to be sitting reasonably well between words and sentences, being closer to sentence unit reading overall. However, the phrase unit condition is a ‘strangely silent’ condition, revealing no effects in means, distributional slopes, or simple recurrence analysis. Furthermore, the patterns of effects observed in cross-recurrence analysis do not mimic the findings from sentence unit reading: %LAM and %DET increase for graduate readers with repeated reading, while %DET decreases for undergraduate readers with repeated reading. This might suggest that graduate students’ reading entrains more strongly to features of the text, which might guide their reading performance, while undergraduates’ performance is less attached to overarching orderly features of the text. Just as with sentence units, multifractal dynamics are basically absent and the monofractal exponents are close to white noise. This suggests that phrase units are much more constraining of the button press performance and likely reflect reading times much better than word unit button presses.

Perhaps the close reading of phrases is necessary because phrases are more ambiguous than sentences – i.e. reading the phrase “Drakh knew it meant time away from his family,” provides a basis to foster expectations about what will follow, but is by no means uniquely predictive of content or length of the phrase that will follow. Also the practical meaning of the phrase depends on what follows thereafter (e.g. time away from his family could be something joyful, sorrowful, or simply unimportant). Garden-path sentences are a prominent example of such “feedback-loops” in text comprehension, where ambiguity in one part of a sentence is resolved through a word or phrase that comes late in the sentence [69]. Sentence surprise endings might perturb on-going comprehension of the story, when presented in phrase units, requiring a closer reading in self-paced phrase unit conditions (of course, dependencies between sentences exist as well, which are the basis for higher order story structure – however, sentences do also have more of a capacity to stand for themselves, providing more a closure of meaning compared to phrases). The somewhat non-optimal parsing by phrases could thus be similar to suboptimal visual parsing of text units shown by LeVassuer and colleagues [67], [71], which was not detrimental to comprehension, but required greater effort on the side of the reader.

Throughout the different measures and statistics, the sentence unit condition distinguishes graduate student and undergraduate readers in fluency. Sentence units are most sensitive to the differences between reader groups, but also to effects of repeated reading. Just as for phrase units, mono- and multifractal statistics suggest that reading performance is much more constrained by these text units, that is what they have in common. However, and in contrast to phrase units, sentence units partly corroborate the picture we get of repeated reading from word units, with shallower distributional tails and decreased in variability, indicating that repeated reading is much about the reduction of surprises a reader encounters. Also, expected drop in reading time with repeated readings was observed in the sentence unit condition, which was masked in the word unit conditions by the delimiting effect of button press speed, and which was masked in the phrase unit condition by, perhaps, the somewhat unfortunate grouping that phrase units provide form the perspective of the reader. Cross recurrence finally revealed the differences between undergraduate and graduate readers across repeated readings, which we interpret as differences in reading fluency and which we will discuss in more detail in the next section.

However, while we could show that single words seem to be problematic to assess adult reading, we do not claim that sentence unit conditions truly replicate the standard reading conditions of continuous text, or reading in any other context other than self-paced reading of sentence units either. Maybe sentence units will prove better to distinguish adult fluency, but it could, for example, turn out that word units distinguish fluency in beginning readers better, for whom correct word reading is still a challenge in itself, and the reading process is so slow, that button presses in the word unit condition might very well reflect actual reading times [19].

### Reading Fluency

Fluency of reading implies effortlessness and flexibility, and of course comprehension and speed [18], [72]. Since all of our participants showed a sufficient level of comprehension, we will start our discussion of fluency based on our observations of sentence-by-sentence reading. For the present study (i.e., literate adult readers that read an easy text), the sentence unit condition seems to show the clearest effects of reading fluency. First of all, reading times decrease with repeated reading for less fluent undergraduate readers, while the more fluent graduate readers showed no change in central tendency (as evidenced by the mode). Also, both reader groups produced more tightly dispersed reading time distributions with repeated reading. So while undergraduate had a tangible speed-performance gain, both groups showed signs of fine-tuning of performance, producing less deviant responses as a consequence of familiarity with the text.

When we look at the dynamic features of reading time performance through cross-recurrence analysis, we see that there is little change in the shared dynamics of reading times for graduate readers, but undergraduates’ reading times show increases in shared %DET and %LAM, as well as a trend for a greater increase in %REC. Especially for %REC and %LAM it is obvious that these gains are toward the level observed in graduate student readers, indicating a capacity for change in performance that toward a greater level of expertise with repeated reading. What is interesting in this context is that the increases in %REC, %DET and %LAM do not indicate overall greater stability or uniformity in performance, as these measures do not appreciably change in individual recurrence analysis. What they rather seem to indicate is that the commonalities in the evolution of the reading performance increase with repeated reading for undergraduates (and stay stable on a high level for graduates) – that is the readers’ performance becomes more tightly coupled to the text. Hence, the process of fluent reading might be one where readers offload the demands of the reading task in parts to the text, letting stimulus drive action, which then perhaps leads to gains in effortlessness on the side of the readers. With this basic picture in mind, two questions arise: First, how are the observed reading patterns of the other two conditions to be interpreted in terms of fluency? Second, what are the relevant features of the text or the text-reader interaction that drive the observed increase in the commonalities between reading performances?

If we take the sentence unit condition as a standard, we see two interesting aspects of fluency in phrase unit reading: One is that gains in reading fluency with repeated reading (shown by an increase in commonalities of temporal evolution of reading times) are observed for the more fluent graduate readers, not for undergraduates. The other one is the generally insensitivity of the phrase unit condition to produce contrasts between the reader groups and the repeated readings. This insensitivity can be interpreted as a perturbation effect of phrase units on the reading performance. As we have pointed out, suboptimal visual parsing of text can hamper reading fluency [70], [71], and it is under these more challenging conditions that we see and improvement of reading ease with repeated reading in graduate readers. That we do not see a similar pattern in undergraduate readers might imply that below a certain level of reading skill, participants find it hard to use the experience of a single repetition of the text to optimize their reading process. However, the observed drop in shared %DET for undergraduates with repeated reading in the phrase unit condition might also imply that less fluent readers use a strategy that is different altogether, and does not hinge on simply offloading aspects of reading performance onto the structure of the reading task.

Finally, word unit reading reveals gains in fluency with repeated reading as a decrease in the temporal commonalities of reading times (as seen in lower shared %REC, %DET and %LAM cross-recurrence), as well as by an loss in structure in reading time series (as seen in lower %DET and %LAM in individual recurrence). We have interpreted this as a shift from partial dependency of the key presses on the text in the first reading to a mainly motor-performance task in the second reading. This also leads from more dispersed reading times that include the partial text-dependencies of the performance to a smoother and less dispersed motor-execution. The general tension between the overall fast reading ability and the comparatively slow word-revealing ability masks any difference between the reader groups, however, and seem to make the word unit condition somewhat suboptimal for the investigations of differences in adult reading performance (but see [19], who could show differences in word unit presentation between groups of beginning readers that were in line with the effects we observed in adults during sentence unit reading).

### Conclusion and Outlook

In sum, our study of habitual and text-dependent fluency in text reading shows that the development of reading fluency – the pinnacle of reading ability – continues beyond the acquisition of literacy and beyond adolescence. Considering the reading *process*, stability of reading time fluctuations, as well as the degree of their coupling to the text are hallmarks of high reading fluency. However, future research is needed to conclude which properties of a text – or which level of a text – are of relevance here, since the lexical feature of the text contributed only minimally to reading performance. Furthermore, we found that for self-paced reading, stimulus presentation on a word-by-word basis was suboptimal, confounding reading skill with participants’ ability of response execution. Sentence-by-sentence presentations seemed a more sensible unit of text presentation for adult readers. These results have been mainly brought about by the application of nonlinear analysis techniques to quantify aspects of the text reading process, where investigations of substantial connected text reading performance has previously been regarded as unfeasible. These techniques carve out promising new avenues for research that involves increasingly complex language materials.

## References

[1] Rawlinson GE (1976) The significance of letter position in word recognition. PhD Thesis. Nottingham: Psychology Department, University of Nottingham.

[2] Epelboim J, Booth JR, Ashkenazy R, Taleghani A, Steinman RM (1997) Fillers and spaces in text: The importance of word recognition during reading. Vision Res 37: 2899–2914. 9415369 10.1016/s0042-6989(97)00095-3

[3] Luce PA, Cluff MS (1998) Delayed commitment in spoken word recognition: evidence from cross-modal priming. Percept Psychophys 60: 484–490. 9599997 10.3758/bf03206868

[4] Rayner K, Pollatsek A (1994) The psychology of reading. Englewood Cliffs, NJ: Prentice Hall.

[5] Clifton C Jr, Duffy SA (2001) Sentence and text comprehension: Roles of linguistic structure. Annu Rev Psychol 52: 167–196. 11148303 10.1146/annurev.psych.52.1.167

[6] Neely JH, VerWys CA, Kahan TA (1998) Reading ‘glasses’ will prime ‘vision’, but reading a pair of ‘glasses’ will not. Mem Cognit 26: 34–39. 10.3758/bf03211368 9519695

[7] Balota DA, Paul ST (1996) Summation of activation: Evidence from multiple primes that converge and diverge within semantic memory. J Exp Psychol Learn Mem Cogn 22: 827–845. 8708602 10.1037//0278-7393.22.4.827

[8] Bargh JA (2006) What have we been priming all these years? On the development, mechanisms, and ecology of nonconscious social behavior. Eur J Soc Psychol 36: 147–168. 19844598 10.1002/ejsp.336 PMC2763379

[9] Pickering MJ, Ferreira VS (2008) Structural priming: A critical review. Psychol Bull 134: 427–459. 18444704 10.1037/0033-2909.134.3.427 PMC2657366

[10] Van Orden G, Kloos H (2005) The question of phonology and reading. In: Snowling M, Hulme C, editors. The Science of Reading: A Handbook. Oxford: Blackwell Publishing.

[11] Fisher DF, Shebilske WL (1985) There is more that meets the eye than the eyemind assumption. In: Groner R, McConkie GW, Menz C, editors. Eye movements and human information processing. Amsterdam: North Holland. 149–158.

[12] Kliegl R, Olson RK, Davidson BJ (1982) Regression analyses as a tool for studying reading processes: Comments on Just and Carpenter's eye fixation theory. Mem Cognit 10: 287–296. 10.3758/bf03197640 7121249

[13] Carroll DW (2003) The psychology of language. Belmont: Thompson.

[14] Bresnan J (2001) Lexical-functional syntax. Oxford: Blackwell Publishing.

[15] Just MA, Carpenter PA (1980) A theory of reading: From eye fixations to comprehension. Psychol Rev 87: 329–354. 7413885

[16] Zwaan RA, Magliano JP, Graesser AC (1995) Dimensions of situation model construction in narrative comprehension. J Exp Psychol Learn Mem Cogn 21: 386–397.

[17] Riley MA, Van Orden G (2005) Contemporary nonlinear methods for behavioral sciences: A webbook tutorial. Available: http://www.nsf.gov/sbe/bcs/pac/nmbs/nmbs.jep. Accessed 23 August 2009.

[18] Wallot S, Van Orden G (2011) Nonlinear analyses of self-paced reading. Ment Lex 6: 245–274.

[19] Wallot S, O’Brien BA, Van Orden G (2012) Fractal and recurrence analysis of psycholinguistic data. In: Westbury C, Jarema G, Libben G, editors. Methodological and Analytic Frontiers in Lexical Research. John Benjamins: Amsterdam. 395–430.

[20] Wallot S, Van Orden G (2011) Toward a lifespan metric of reading fluency. Int J Bifurcation Chaos 21: 1173–1192.

[21] Samuels SJ (1979) The method of repeated readings. Read Teach 32: 403–408.

[22] DeGrado LP (2003) The Arelis complex. Available: http://www.authorsden.com/visit/viewshortstory.asp?id=9632. Accessed 23 August 2009.

[23] Fengxiang F (2007) A corpus based quantitative study on the change of TTR, word length and sentence length of the english language. In: Grzybek P, Kohler R, editors. Exact methods in the study of language and text: Dedicated to Gabriel Altmann on the occasion of his 75th birthday. Berlin: de Gruyter. 123–130.

[24] Sigurd B, Eeg-Olofsson M, van de Weijer J (2004) Word length, sentence length, and frequency–Zipf revisited. Studia Linguistica 58: 37–52.

[25] Oakland T, Lane H (2004) Language, Reading, and Readability Formulas: Implications for Developing and Adapting Tests. Int J Test 4: 239–252.

[26] Brainard DH (1997) The psychophysics toolbox. Spat Vis 10: 433–436. 9176952

[27] Rasinski TV (2012) Why reading fluency should be hot. The Reading Teacher 65: 516–522.

[28] Engel BT, Thorne PR, Quilter RE (1972) On the relationship among sex, age, response mode, cardiac cycle phase, breathing cycle phase, and simple reaction time. J Gerontol 27: 456–460. 5075489 10.1093/geronj/27.4.456

[29] Balota DA, Yap MJ, Cortese MJ, Hutchison KA, Kessler B, et al (2007) The English lexicon project. Behav Res Methods 39: 445–459. 17958156 10.3758/bf03193014

[30] Just MA, Carpenter PA, Woolley JD (1982) Paradigms and processes in reading comprehension. J Exp Psychol Gen 111: 228–238. 6213735 10.1037//0096-3445.111.2.228

[31] Newell KM, Liu Y-T, Mayer-Kress G (2001) Time scales in motor learning and development. Psychol Rev 108: 57–82. 11212633 10.1037/0033-295x.108.1.57

[32] van Rooij MMJW, Nash B, Rajaraman S, Holden JG (2013) A fractal approach to dynamic inference and distribution analysis. Front Physiol: doi: 10.3389/fphys.2013.00001. 10.3389/fphys.2013.00001 PMC3557596 23372552

[33] Holden JG, Van Orden G, Turvey MT (2009) Dispersion of response times reveals cognitive dynamics. Psychol Rev 116: 318–342. 19348544 10.1037/a0014849 PMC3730291

[34] Brown C, Liebovitch L (2010) Fractal analysis. London: Sage.

[35] Frank SL, Bod R (2011) Insensitivity of the human sentence-processing system to hierarchical structure. Psychol Sci: doi:10.1177/0956797611409589. 10.1177/0956797611409589 21586764

[36] Kennedy A, Pynte J (2005) Parafoveal-on-foveal effects in normal reading. Vision Res 45: 153–168. 15581917 10.1016/j.visres.2004.07.037

[37] Graf R, Nagler M, Jacobs AM (2005) Faktorenanalyse von 57 Variablen der visuellen Worterkennung (factor analysis of 57 variables in visual word recognition). Z Psychol 213: 205–218.

[38] Shaoul C, Westbury C (2006) USENET orthographic frequencies for the 40,481 words in the english lexicon project. Available: www.psych.ualberta.ca/~westburylab/downloads/elp.download.html. Accessed 7 July 2011.

[39] Landauer TK, Foltz PW, Laham D (1998) An introduction to Latent Semantic Analysis. Discourse Process 25: 259–284.

[40] Lund K, Burgess C (1996) Producing high-dimensional semantic spaces from lexical co-occurrence. Behav Res Methods 28: 203–208.

[41] Landauer TK, Dumais ST (1997) A solution to Plato’s problem: The Latent Semantic Analysis theory of the acquisition, induction, and representation of knowledge. Psychol Rev 104: 211–240.

[42] Laham D (1998) Latent Semantic Analysis @ CU Boulder. Available: http://lsa.colorado.edu/. Accessed 8 August 2012.

[43] Baayen RH (2010) Demythologizing the word frequency effect. Ment Lex 5: 436–461.

[44] Cordier F, Croizet J-C, Rigalleau F (2012) Comparing nouns and verbs in a lexical task. J Psycholinguist Res: doi:10.1007/s10936-012-9202-x. 10.1007/s10936-012-9202-x 22415732

[45] Orsucci F, Giuliani A, Webber C, Zbilut J, Fonagy P, et al (2006) Combinatorics and synchronization in natural semiotics. Physica A 361: 665–676.

[46] Webber CL Jr, Zbilut JP (2005) Recurrence quantification analysis of nonlinear dynamical systems. In: Accessed 23 August Riley MA, Van Orden G, editors. Contemporary nonlinear methods for behavioral sciences: A webbook tutorial. Available: http://www.nsf.gov/sbe/bcs/pac/nmbs/nmbs.jep. Accessed 23 August 2009.pp. 26–96.

[47] Holden JG (2005) Gauging the fractal dimension of response times from cognitive tasks. In Riley MA, Van Orden G, editors. Contemporary nonlinear methods for behavioral sciences: A webbook tutorial. 267–318. Available: http://www.nsf.gov/sbe/bcs/pac/nmbs/nmbs.jep. Accessed 23 August 2009.

[48] Marwan N, Kurths J, Thomsen JS, Felsenberg D, Saparin P (2009) Three dimensional quantification of structures in trabecular bone using measures of complexity. Phys Rev E: doi:10.1103/PhysRevE.79.021903. 10.1103/PhysRevE.79.021903 19391774

[49] Webber CL Jr, Zbilut JP (1994) Dynamical assessment of physiological systems and states using recurrence plot strategies. J Appl Physiol 76: 965–973. 8175612 10.1152/jappl.1994.76.2.965

[50] Stephen DG, Dixon JA, Isenhower RW (2009) Dynamics of representational change: Entropy, action, and cognition. J Exp Psychol: Hum Percept Perform 35: 1811–1822. 19968438 10.1037/a0014510

[51] Wijnants ML, Hasselman F, Cox RFA, Bosman AMT, Van Orden G (2012) An interaction-dominant perspective on reading fluency and dyslexia. Ann Dyslexia 62: 100–119. 22460607 10.1007/s11881-012-0067-3 PMC3360848

[52] Kuznetsov NA, Riley MA (2011) Spatial resolution of visual feedback affects variability and structure of isometric force. Neurosci Lett 470: 121–125. 10.1016/j.neulet.2009.12.068 20045718

[53] Schmit JM, Regis D, Riley MA (2005) Dynamic patterns of postural sway in ballet dancers and track athletes. Exp Brain Res 163: 370–378. 15655686 10.1007/s00221-004-2185-6

[54] Konvalinka I, Xygalatas D, Bulbulia J, Schjødt U, Jegindø EM, et al.. (2011) Synchronized arousal between performers and related spectators in a fire-walking ritual. Proc Natl Acad Sci U S A: doi: 10.1073/pnas.1016955108. 10.1073/pnas.1016955108 PMC3100954 21536887

[55] Takens F (1981) Detecting strange attractors in turbulence. Lecture Notes in Mathematics 898: 366–381.

[56] Marwan N (2011) How to avoid potential pitfalls in recurrence plot based data analysis? Int J Bifurcat Chaos 21: 1003–1017.

[57] Shockley K, Butwill M, Zbilut J, Webber CL Jr (2002) Cross recurrence quantification of coupled oscillators. Phys Lett A 305: 59–69.

[58] Shockley K (2005) Cross recurrence quantification of interpersonal postural activity. In: Accessed 23 August Riley MA, Van Orden G, editors. Contemporary nonlinear methods for behavioral sciences: A webbook tutorial. Available: http://www.nsf.gov/sbe/bcs/pac/nmbs/nmbs.jep. 2009: 26–96.

[59] Kloos H, Van Orden G (2010) Voluntary behavior in cognitive and motor tasks. Mind & Matter 8: 19–43.

[60] Van Orden G, Kloos H, Wallot S (2011) Living in the Pink: Intentionality, Wellbeing, and Complexity. In: Hooker CA, editor. Philosophy of Complex Systems. Handbook of the Philosophy of Science. Amsterdam: Elsevier. 639–684.

[61] Wijnants ML, Bosman AMT, Hasselman F, Cox RFA, Van Orden G (2009) 1/f scaling in movement time changes with practice in precision aiming. Nonlinear Dynamics Psychol Life Sci 13: 79–98. 19061546

[62] McAuley JD, Jones MR, Holub S, Johnston HM, Miller NS (2006) The time of our lives: Lifespan development of timing and event tracking. J Exp Psychol Gen 135: 348–367. 16846269 10.1037/0096-3445.135.3.348

[63] Chen Y, Ding M, Kelso JAS (1997) Long term memory processes (1/fα type) in human coordination. Phys Rev Lett 79: 4501–4504.

[64] Deligniéres D, Torre K, Lemoine L (2009) Long-range correlation in synchronization and syncopation tapping: A linear phase correction model. PLoS ONE: doi:10.1371/journal.pone.0007822. 10.1371/journal.pone.0007822 PMC2771896 19915658

[65] Ihlen EAF, Vereijken B (2010) Interaction-dominant dynamics in human cognition: Beyond 1/fα fluctuation. J Exp Psychol Gen 139: 426–463. 10.1037/a0019098 20677894

[66] Gibbs RW Jr (1994) The poetics of the mind: Figurative thought, language, and understanding. New York: Cambridge University Press.

[67] Wengelin Å (2006) Examining pauses in writing: Theory, methods and empirical data. In: Sullivan K, Lindgren E, editors. Computer key-stroke logging and writing: methods and applications. Amsterdam: Elsevier. 107–130.

[68] Wallot S, Grabowski J (2013) Typewriting Dynamics: What Distinguishes Simple from Complex Writing Tasks? Ecol Psychol. In press.

[69] Frazier L, Rayner K (1982) Making and correcting errors during sentence comprehension: Eye movements in the analysis of structurally ambiguous sentences. Cognit Psychol 14: 178–210.

[70] LeVasseur VM, Macaruso P, Shankweiler D (2008) Promotion gains in reading fluency: a comparison of three approaches. Read Writ 21: 205–230.

[71] LeVasseur VM, Macaruso P, Palumbo LC, Shankweiler D (2006) Syntactically cued text facilitates oral reading fluency in developing readers. Appl Psycholinguist 27: 423–445.

[72] Meyer MS, Felton RH (1999) Repeated reading to enhance fluency: Old approaches and new directions. Ann Dyslexia 49: 283–306.

